# Preferential graphitic-nitrogen formation in pyridine-extended graphene nanoribbons

**DOI:** 10.1038/s42004-024-01344-7

**Published:** 2024-11-21

**Authors:** Nicolò Bassi, Xiushang Xu, Feifei Xiang, Nils Krane, Carlo A. Pignedoli, Akimitsu Narita, Roman Fasel, Pascal Ruffieux

**Affiliations:** 1 nanotech@surfaces Laboratory, Empa, Swiss Federal Laboratories for Materials Science and Technology, Dübendorf, Switzerland; 2 Organic and Carbon Nanomaterials Unit, Okinawa Institute of Science and Technology Graduate University, Okinawa, Japan; 3 Department of Chemistry, Biochemistry and Pharmaceutical Sciences, University of Bern, Bern, Switzerland

**Keywords:** Electronic properties and devices, Scanning probe microscopy, Surface spectroscopy

## Abstract

Graphene nanoribbons (GNRs), nanometer-wide strips of graphene, have garnered significant attention due to their tunable electronic and magnetic properties arising from quantum confinement. A promising approach to manipulate their electronic characteristics involves substituting carbon with heteroatoms, such as nitrogen, with different effects predicted depending on their position. In this study, we present the extension of the edges of 7-atom-wide armchair graphene nanoribbons (7-AGNRs) with pyridine rings, achieved on a Au(111) surface via on-surface synthesis. High-resolution structural characterization confirms the targeted structure, showcasing the predominant formation of carbon-nitrogen (C-N) bonds (over 90% of the units) during growth. This favored bond formation pathway is elucidated and confirmed through density functional theory (DFT) simulations. Furthermore, an analysis of the electronic properties reveals metallic behavior due to charge transfer to the Au(111) substrate accompanied by the presence of nitrogen-localized states. Our results underscore the successful formation of C-N bonds on the metal surface, providing insights for designing new GNRs that incorporate substitutional nitrogen atoms to precisely control their electronic properties.

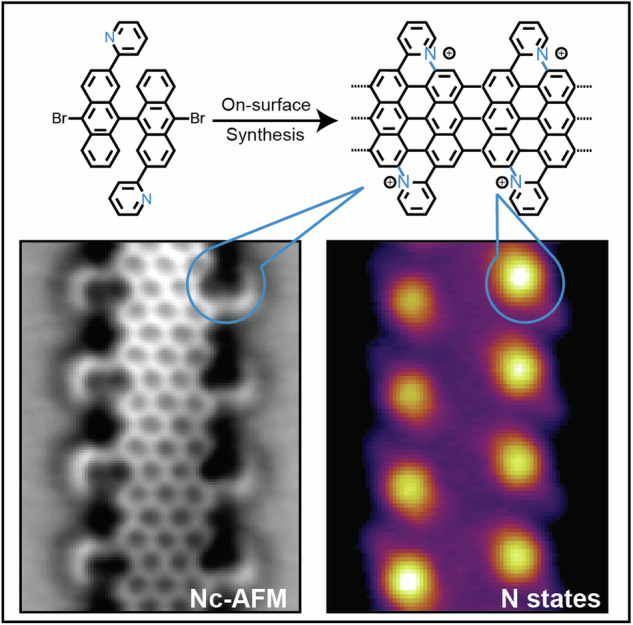

## Abstract (web summary)

The presence of graphitic nitrogen atoms within graphene nanoribbons has been predicted to strongly affect their electronic properties, but its experimental formation within such structures remains challenging. Here, the authors report on the on-surface synthesis of pyridine-extended 7-armchair graphene nanoribbons on Au(111), whereby graphitic nitrogen is preferentially formed after complete planarization through the formation of C–N bonds.

## Introduction

Graphene nanoribbons (GNRs), one-dimensional graphene stripes with nanometer width, represent a distinctive class of carbon materials characterized by a band gap from quantum confinement. Depending on the geometry and edge terminations, GNRs can be classified into various groups such as: Armchair (AGNRs)^1–5^, Zigzag (ZGNRs)^6,7^, Chevron^1,8–10^ and other structures^11^. The ability to fine-tune their electronic properties by altering the chemical structure is a pivotal aspect driving extensive research; for example, the band gap of AGNRs can be controlled by adjusting the ribbon width^12–14^.

Introducing substitutions of C atoms with elements from neighboring groups of the periodic table adds further degrees of freedom, enabling “doping” in GNRs to precisely modulate their electronic properties. Consequently, significant attention has been devoted to exploring GNRs substituted with elements like Oxygen^15,16^, Sulfur^15,17–19^, Boron^20,21^ and Nitrogen^7,22–33^; either individually or in combination^34^. Nitrogen-substitution, in particular, has been deeply investigated due to its ability to act as an electron donor in GNRs.

The local chemical environment of the substituted Nitrogen leads to various electronic configurations in graphene-related structures^35,36^. For instance, *pyridinic-*N contributes only one electron to the π-states because of its *-sp*^2^ hybridization and the bonds with two neighboring atoms, i.e., it behaves similar to a CH group^37^. It is found at the edges of the GNRs and causes a rigid downshift of valence (VB) and conduction bands (CB) which has been exploited to create local *p-n* junctions in GNRs^7,22,38,39^. On the other end, *graphitic-*N with three bonds to neighboring C atoms and an extra electron partially shared with the π orbitals^37,40^ reduces the band gap and leads to the formation of a new in-gap state localized at the heteroatom. However, the formation of *graphitic-*N in GNRs is less common, with few recent examples^30,34,41^.

Fine-tuning the electronic properties of GNRs demands precise structural control down to the atomic level, a challenge not met by modern top-down techniques. On-surface synthesis has emerged as a promising bottom-up approach to achieve this precision^1,13,42^. This synthesis approach involves the surface-assisted activation of precursors for polymer growth through Ullmann-type coupling and subsequent higher temperature cyclodehydrogenation to planarize the polymers. In most of the GNRs investigated so far, only C and H were involved in the different synthetic steps. On-surface synthesis has also been applied to doped structures where, however, the substituting elements generally are not involved in the formation of new bonds^22,29,38^ with very few exceptions^43,44^.

Here, we present the on-surface synthesis of a pyridine-extended 7-AGNR (Py-7-AGNR) on Au(111) from 10,10’-dibromo-2,2’-di(pyridin-2-yl)-9,9’-bianthryl (DB-DPBA) and investigate its properties using scanning tunneling microscopy (STM) and non-contact atomic force microscopy (nc-AFM). We find preferential bond formation between the N of the pyridine extension and the 7-AGNR backbone (>90%) resulting in the integration of *graphitic-*N along the GNR edge. The other possible pyridine orientation, leading in C-C bonding and formation of *pyridinic-*N, is largely suppressed. This preferential C-N versus C-C reaction pathways is rationalized by a lower activation barrier, as determined from DFT calculations. The impact of *graphitic-*N on the electronic properties of Py-7-AGNRs is probed by means of scanning tunneling spectroscopy (STS) revealing a reduction in the band gap and the emergence of localized states in agreement with DFT simulations.

## Results and discussions

**Fig. 1:**
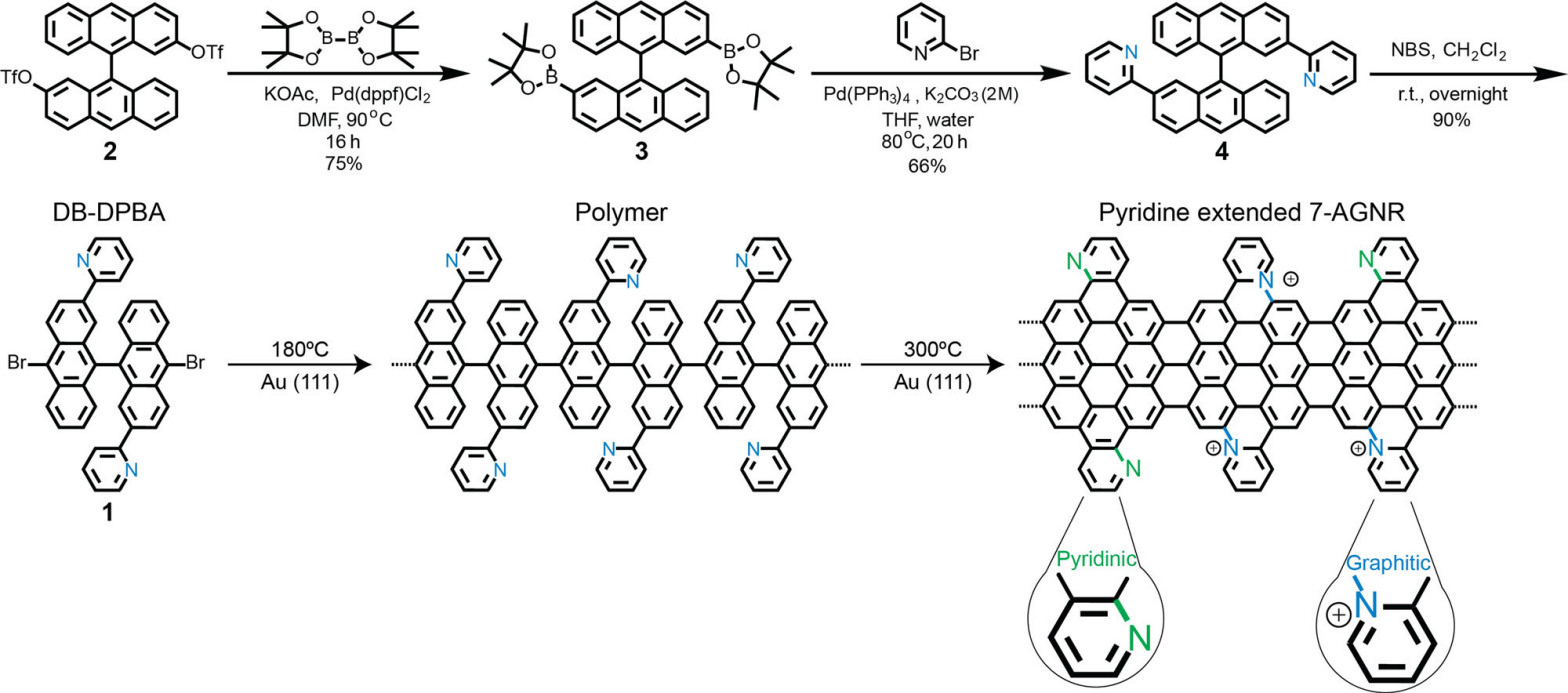
Synthesis steps for Py-7-AGNRs. Solution-synthesis of monomer **1** (upper row), used for the on-surface synthesis of Py-7-AGNRs (bottom row). The two possible configurations resulting in *pyridinic*-N and *graphitic-*N are highlighted in the last panel.

The molecular precursor DB-DPBA (**1**) consists of a bianthracene backbone with two pyridyl groups attached at opposite sides and two Br atoms at opposite edges. Precursor (**1**) was synthesized in-solution, as shown in the top row of Fig. 1. Bistriflate (**2**) was initially prepared according to previously reported procedures^45^ and subjected to the Miyaura-Ishiyama borylation to provide diboronic ester (**3**) in 75% yield. Then, the Suzuki-Miyaura coupling of (**3**) and 2-bromopyridine afforded 2,2’-di(pyridin-2-yl)-9,9’- bianthracene (**4**) in 66% yield. Finally, DB-DPBA **1** was obtained by the bromination of (**4**) using N-bromosuccinimide (NBS) at room temperature in 90% yield and characterized by ^1^H and ^13^C NMR spectroscopy as well as high-resolution mass spectrometry (see SI 1-6 in the Supporting Information).

Subsequently, precursor **1** was sublimation-deposited on a clean Au(111) substrate in ultra-high vacuum (UHV) to perform on-surface reactions. A first annealing step at around 180 °C triggers the Ullmann-type coupling between DB-DPBA precursors to form non-planar polymers. A second annealing step at 300 °C induces complete planarization to form the targeted Py-7-AGNR. Due to the asymmetry in the lateral pyridyl units and their possible rotations during the annealing process, the positions of the N atoms along the ribbon are not predetermined. In particular, the pyridyl group can cyclize under formation of either a C-N or a C-C bond, leading to a *graphitic-*N or a *pyridinic-*N (highlighted in blue and green, respectively, in Scheme 1). To understand if one of the two possible N configurations is favored, we deposited a sub-monolayer coverage of DB-DPBA on a Au(111) substrate kept at room temperature. Recently, Mugarza et al. synthesized a closely related pyridyl-extended precursors with N atoms in 3 or 4 position; however, they reported that sublimation of these precursors was impossible since they already react and polymerize in the crucible^46^. We did not face this problem with DB-DPBA **1**, indicating that the position of the substituted atoms can greatly affect the chemical reactivity of this kind of precursors.

**Fig. 2:**
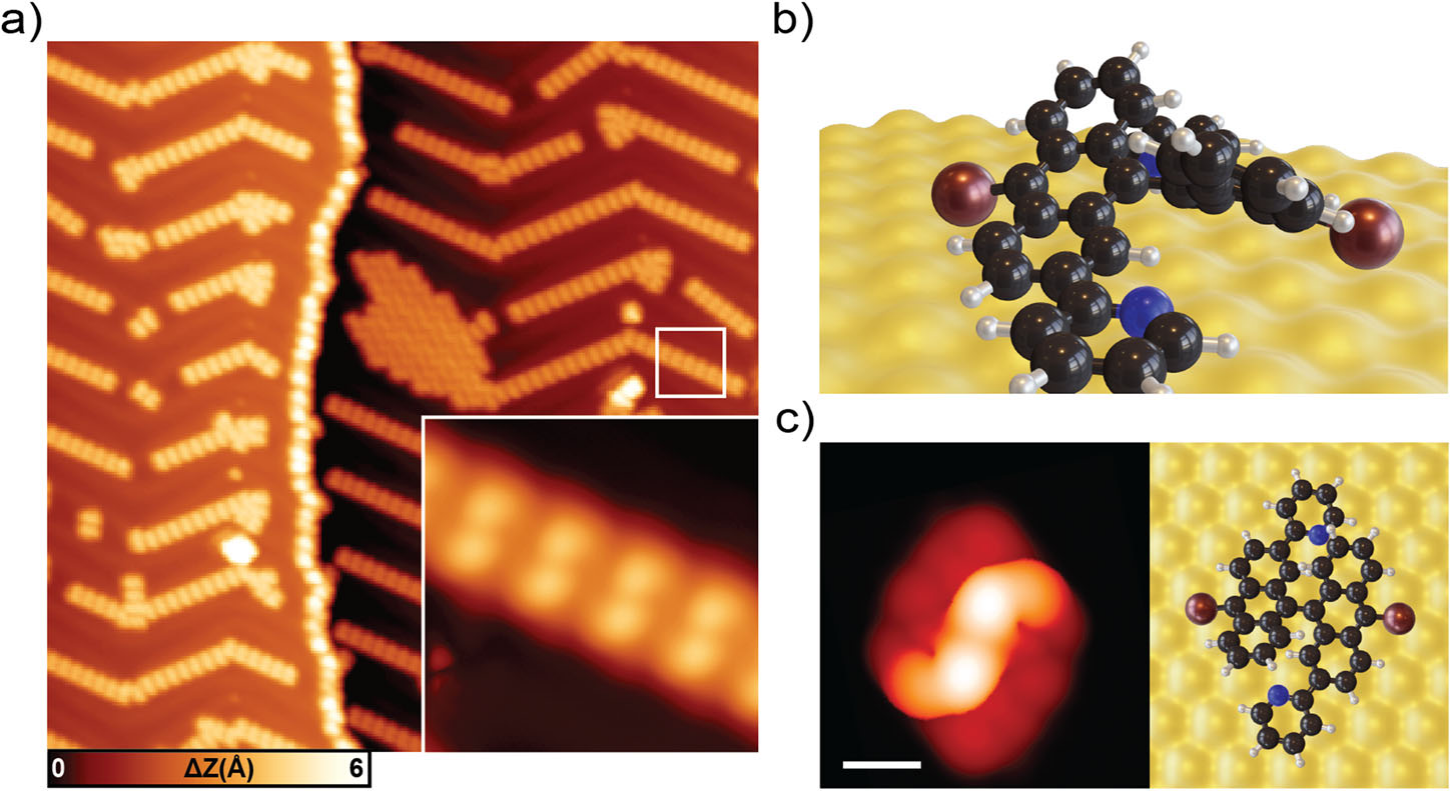
Self-assembly of DB-DPBA on Au(111). **a** Large-scale STM topography after deposition of the molecular precursor. The molecules tend to organize in chains following the Au(111) herringbone reconstruction (scale bar: 20 nm, scanning parameters: −1 V, 50 pA). Inset: small-scale STM topography of the molecules organized in chains. The DB-DBPA are characterized by two brighter dots. **b** Equilibrium geometry of a single DB-DPBA obtained by DFT calculations (perspective view). The pyridyl units lay on the surface, while the bianthracene core is highly tilted. **c** DFT simulated STM image of a single molecule and top view of the equilibrium geometry (scale bar: 1 nm). There is good a matching with the STM appearance of molecules in the topography image in panel a.

A large-scale STM topography image of the surface after DB-DPBA deposition is shown in Fig. 2a. The molecules tend to self-assemble into one-dimensional (1-D) chains at the fcc regions of the herringbone reconstruction of Au(111). Locally, it is possible to find some large islands of close-packed units. The inset of Fig. 2a shows a zoom-in image of a self-assembled chain of molecules. In STM images, the precursors are characterized by two bright, slightly asymmetric features separated by roughly 0.75 nm and an apparent height of 1.9 nm. To elucidate the molecular structure on Au(111), we conduct DFT simulations. Figure 2b shows the relaxed geometry of DB-DPBA on a non-reconstructed Au(111). The precursor adsorbs with the pyridyl lateral groups parallel to the substrate, while the bianthracene backbone is tilted away from the surface, with a maximum height of roughly 0.67 nm. The topmost parts of the two anthracene units have a distance of 0.70 nm, comparable to our experimental values. The corresponding simulated STM image of a DB-DPBA unit (Fig. 2c) matches well with our topography images, where two bright dots are resolved. This adsorption geometry is similar to the one reported for the pristine version (i.e., without N atoms) of the precursor^11,47^.

After deposition, it is not possible to distinguish the position of the N atoms by STM, since all the possible geometries look identical. This is confirmed by DFT-based STM simulation of different pyridyl configurations (see Figure SI 7 and Table 1 in the Supporting Information). The only difference is in terms of adsorption energy, where the structure with the N atoms of the pyridyl pointing outside is favored (−0.11 eV). DFT simulations allow us to estimate the energy barrier needed to flip the pyridyl ring between the two configurations as 0.45 eV at 95°, due to the steric hindrance between the H atoms of the two rings (Fig. SI 8 in the Supporting Info). For larger rotation angles, the energy decreases. Rotation of the pyridyl ring, hence, sees an energy barrier of 0.45 eV and will thus be accessible already at moderate annealing temperatures.

**Fig. 3:**
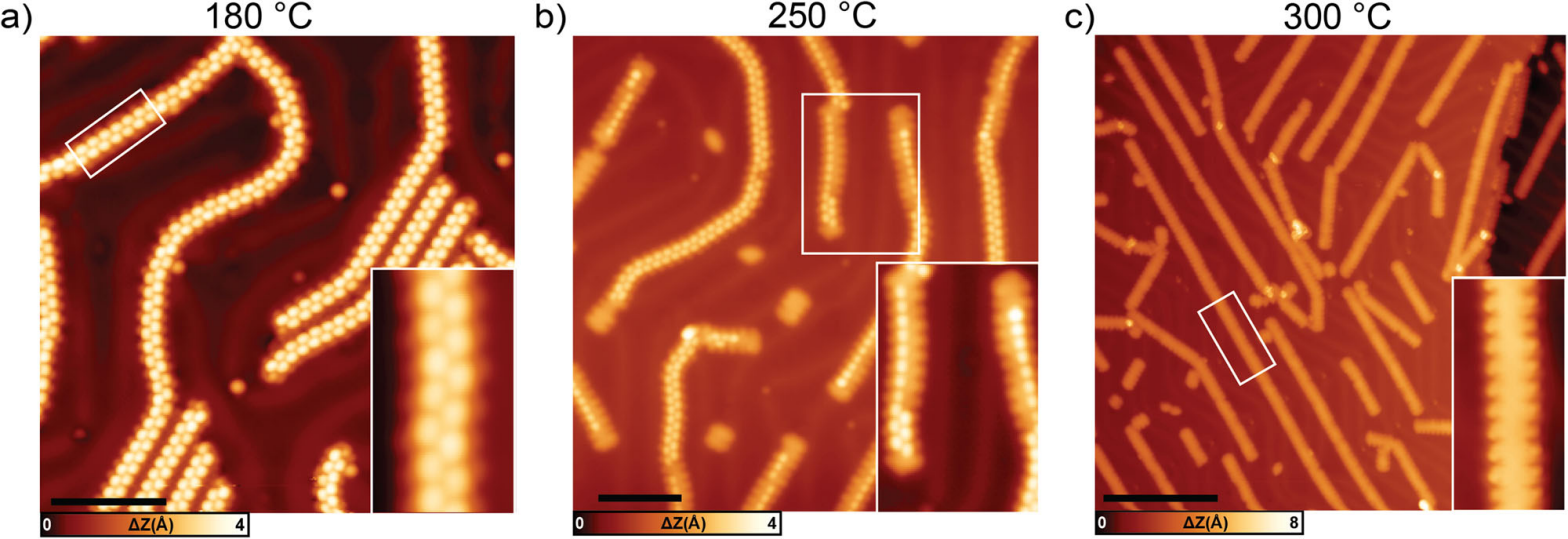
Different annealing temperatures of DB-DBPA on Au(111). **a** Large-scale STM topography after annealing at 180 °C. In the inset, a zoom-in image of a segment of a polymer with alternating dots with a period of 0.85 nm (scale bar: 5 nm, scanning parameters: −0.5 V, 30 pA). **b** Large-scale STM of partially planar segments after annealing at 250 °C. In the inset, a zoom-in image of partially planar segments (scale bar: 10 nm, scanning parameters: −0.5 V, 20 pA. At this temperature, some edges have already undergone planarization. In general, this process starts at the GNR end and proceeds along one edge. **c** Large-scale STM image of planar GNRs at 300 °C. In the inset, a zoom-in image of a segment of a ribbon (scale bar: 10 nm, scanning parameters: −0.3 V, 40 pA.) At this temperature, the segments are already completely planar.

Annealing the sample to 180 °C triggers the formation of polymeric units on the surface, as shown in Fig. 3a. This step results in the growth of polymers (with an average length of 40 nm) which are characterized by bright dots alternating along the main axis with a periodicity of 0.85 nm, as expected from covalently bonded and alternatingly tilted anthracene units. Upon a further annealing step at slightly higher temperature (250 °C) most of the units remain non-planar. However, as shown in the inset of Fig. 3b, some segments have already reacted. This partial planarization process starts at one GNR end and then proceeds along one edge, until a defect (e.g., a bend) is encountered. The DFT-optimized model and the corresponding STM simulation of a partially closed single unit (see Fig. SI 9 in the Supporting Information) matches well with our STM images, where only one brighter dot related to the unreacted edge is seen. Some short units (mainly monomers or dimers) are already completely reacted and planarized.

The final annealing step at a temperature of 300 °C forms completely planar Py-7-AGNRs, as shown in Fig. 3c. These growth conditions can give rise to long (up to 80 nm) and straight GNRs following the elbow sites of the Au(111) reconstruction. Similarly long ribbons have been reported for the pristine version of the same precursor^11,48^; in contrast, bipyrimidine substituted analogues (i.e., two N atoms in each ring) have been reported to grow shorter due to the stronger interaction with the substrate which hinders their diffusion on the surface^46^. Small scale images of the Py-7-AGNR (inset in Fig. 3c), reveal the expected periodic pattern related to the pyridine edge extensions.

In our N-substituted case, complete planarization is reached already at 300 °C instead of 400 °C as in the nitrogen-free 7-AGNR counterpart^11,48^. This lower cyclization temperature is attributed to the presence of N atoms in the pyridyl rings, which is in line with observations in other N-substituted small nanographenes^43,44,46^.

**Fig. 4:**
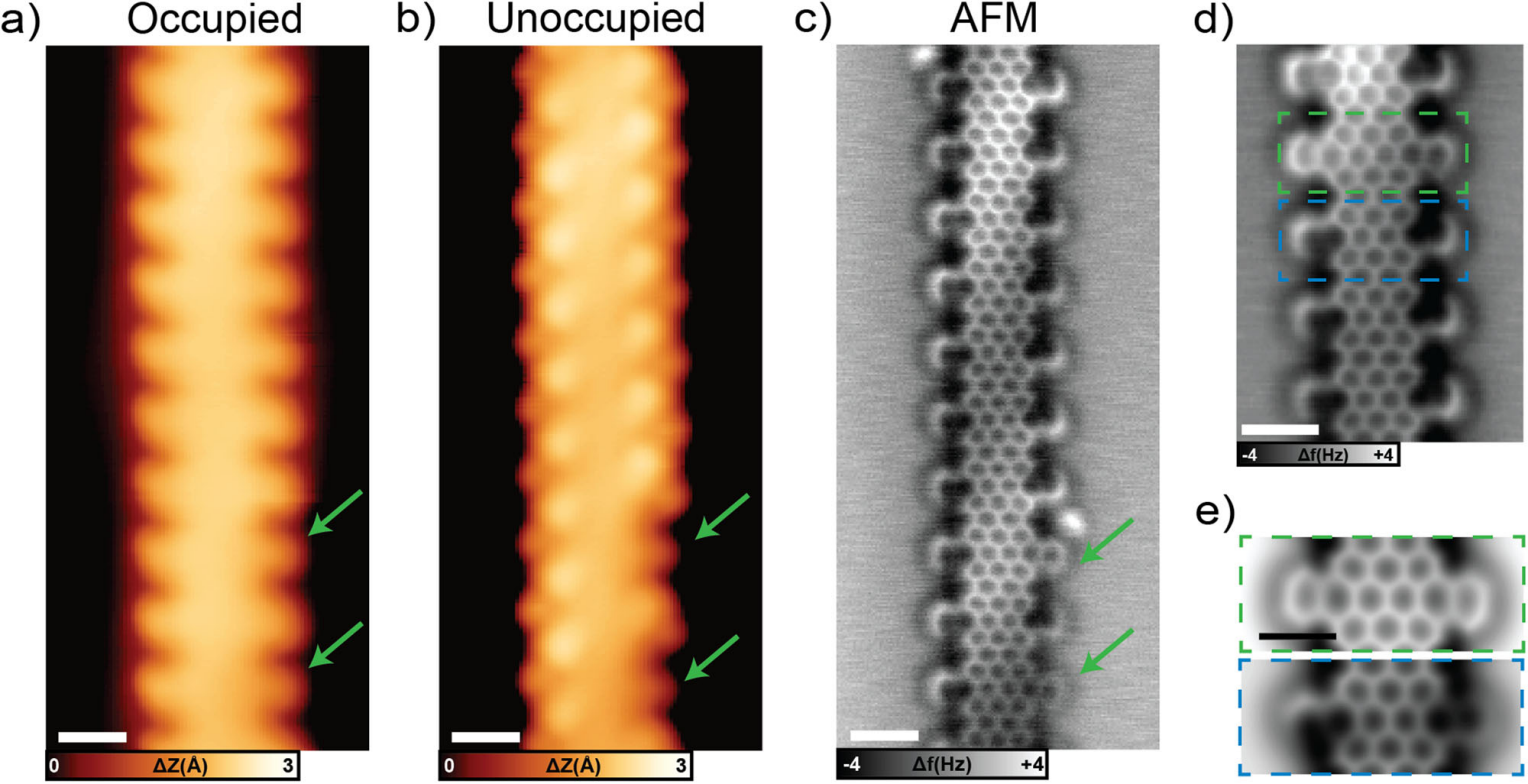
Planar GNRs on Au(111) after annealing at 300 °C. **a** STM topography image acquired in the occupied density of states. Most of the edge extensions have an upward (left side) or downward (right side) bend, with the exception of the two highlighted by green arrows (scale bar: 1 nm, scanning parameters:-1.0 V, 100 pA). **b** STM topography image of the same segment acquired in the unoccupied density of states. The edge extensions are imaged as bright dots, with the exception of the two indicated by green arrows (scale bar: 1 nm, scanning parameters: 0.80 V, 100 pA). **c** Bond-resolved nc-AFM image of the same segment of Py-7-AGNRs, revealing specific bonds in the pyridine extensions that appear darker. (open feedback parameters: −5 mV, 200 pA, Δz: 220 pm from Au(111) surface; frequency: 27401 Hz) (**d**) Nc-AFM image of a short segment of another Py-7-AGNR. The blue dashed box highlights a unit with fully *graphitic-*N edges, while the green box indicates a unit with *pyridinic-*N. (open feedback parameters: −5 mV, 200 pA, Δz: 210 pm from Au(111) surface; frequency: 27401 Hz) (**e**) DFT-based nc-AFM simulations of *pyridinic-*N (green) and *graphitic-*N (blue) units (scale bar: 1 nm).

To better understand if N has a role in the planarization/cyclization steps, we investigated the structure of the synthesized Py-7-AGNR in detail. Figure 4a shows an STM image of a ribbon segment acquired at negative bias (occupied states). Most of the edge extensions seem asymmetric with an apparent “up-bend” on the left and “down-bend” on the right edge with the exception of two units in the bottom right part (highlighted by green arrows) which appear more symmetric and rounded. Measurements taken at positive bias (unoccupied states) (Fig. 4b), instead, show alternating bright dots present along the backbone. The distance between two consecutive brighter units is 1.28 nm with an angle of 63° with respect to the ribbon axis. As for the negative bias image (Fig. 4a), almost all the edges present these features with the exception of the same two highlighted by green arrows.

To understand the reason of this difference, we performed nc-AFM measurements with a CO molecule attached to the tip^49^. In this way, it is possible to achieve single bond resolution, such as in Fig. 4c. Here, the periodic pattern related to the pyridine edge extensions is clearly visible. In addition, the majority of the pyridine extensions reveal bonds that are imaged darker than the ribbon backbone. We attribute these darker bonds to *graphitic-*N-C bonds, and the two brighter ones (highlighted again by green arrows) to *pyridinic-*N-C. Figure 4d shows a high-resolution nc-AFM image of a short segment with one unit (blue rectangle) with edges in *graphitic-*N configuration and the adjacent one (green rectangle) with *pyridinic-*N. Nc-AFM simulations done on the two possible structures (Fig. 4e) clearly confirm our assignment. As expected, *graphitic-*N edges appear dark, while *pyridinic-*N does not have any evident contrast difference, just a slight distortion at the N position. The darker appearance of *graphitic-*N in nc-AFM measurements, already reported for other structures^24,25,50,51^, derives from its different short-range repulsion distance compared to C atoms and the lower adsorption height from the Au substrate (as proven by DFT). In addition, STM simulations of Py-7-AGNR containing *graphitic-*N and *pyridinic-*N (Figure SI 10) reproduce the experimental findings where the extension assigned to *graphitic-*N appears slightly tilted at negative bias while having an apparent bright dot when scanned at around 0.8 eV. *Pyridinic-*N, on the other hand, appears symmetric and rounded.

**Fig. 5:**
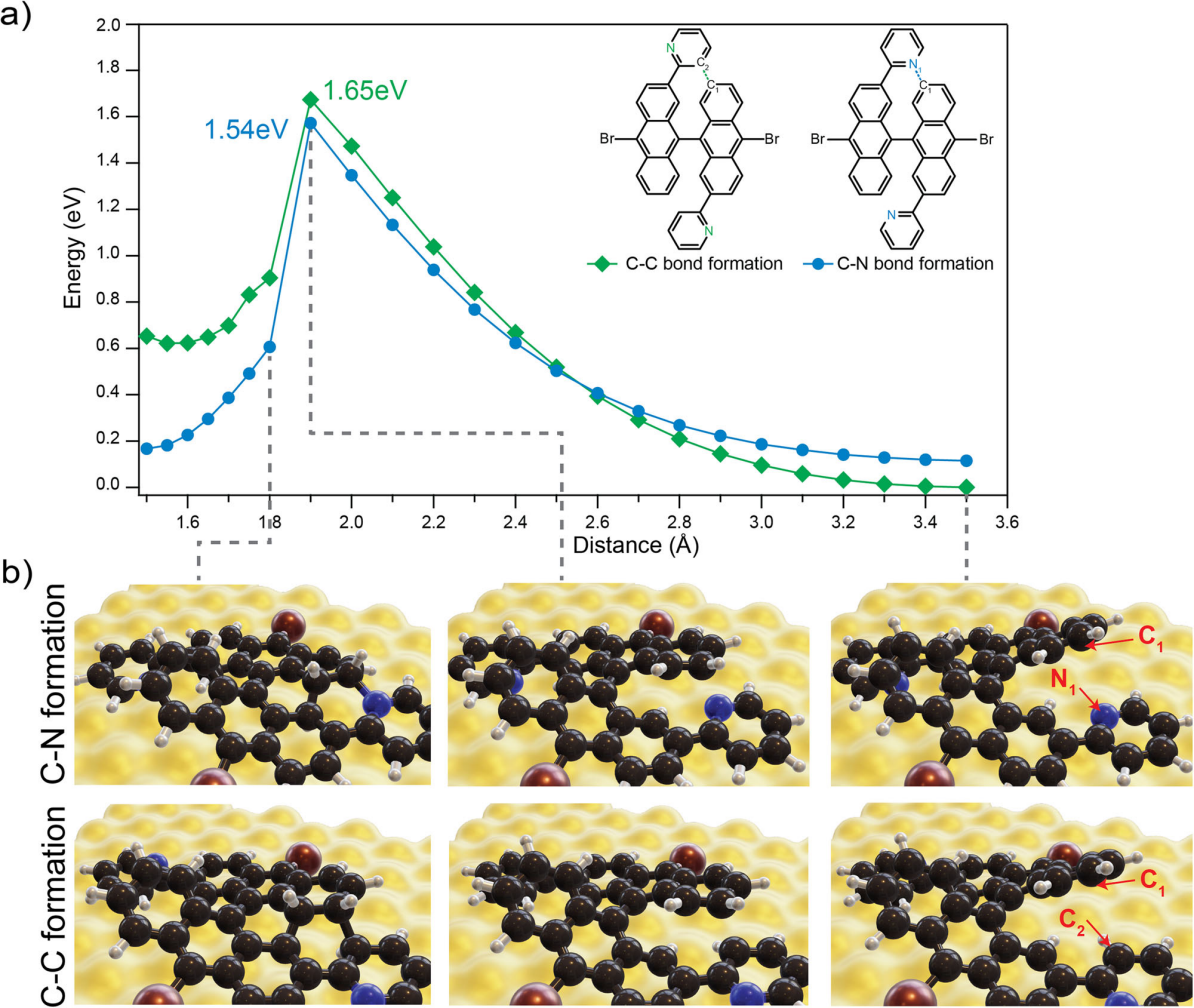
Reaction barrier calculations for C-N and C-C bonds formation. **a** Energy barrier for the C-N (blue) and C-C (green) bond formation as a function of atomic distances. In the inset, chemical sketch with dotted lines indicating the two controlled distances. The energy progressively increases as the distance is reduced until maxima are reached at 1.9 Å. After these points, the two curves decrease in energy thanks to the formation of bonds in the bianthracene backbone. **b** DFT-relaxed structures (perspective view) of different steps of the bond formations. In the right panel, the atoms involved are highlighted with red arrows.

We made statistics on the type of N bonding using nc-AFM images (see also SI 11 in the Supporting Info). From 306 edge extensions that we analyzed, 9.2% were exhibiting a *pyridinic-*N configuration, while all others were *graphitic*-N. Considering the design of the DB-DPBA precursor and the required on-surface synthesis steps implies that cyclization proceeds via preferential C-N bond formation (blue in Scheme 1). Moreover, this ring closure takes place at a lower temperature than the analogous C-C one for the pristine version. For a deeper understanding of this preferred cyclodehydrogenation path, we applied a DFT-based constrained optimization approach to determine the energy barriers for closing the C-N and C-C bonds. As model systems, we used two relaxed geometries of monomers which, upon cyclodehydrogenation, should form a *graphitic-*N or *pyridinic-*N bond (left panels in Fig. 5b). The energy difference of the starting configurations for the C-N *versus* C-C path is due to the different orientation of the pyridyl extension. We used the distance between the two atoms forming a bond as collective variable (atoms connected by dotted lines in the inset of Fig. 5a and indicated with red arrows in Fig. 5b). While slowly changing the constraint value of the collective variable (at increments of 0.05 Å) we relaxed within DFT the geometry (all degrees of freedom except the constraint) thus identifying a transition state (activation energy). Figure 5b shows some configurations of the molecules at each steps.

Figure 5a shows the resulting energy profiles of the two bond formation pathways. Total energies increase as the distance between atoms is reduced, until at 2.6 Å the C-N formation becomes energetically favored. Both energies then reach a maximum at 1.9 Å, but C-N bond formation sees a lower barrier than C-C bond formation. The energy difference of ≈ 0.1 eV comes from the absence of the H atom bonded to the N, which decreases the steric hindrance and the required energy to form the bond. Upon further approach of the atoms, the bonds between the bianthracene units start to form and the energy drops substantially, with the *graphitic-*N case being lower in total energy. The actual mechanism of C-N bond formation has been previously discussed^44^. Applying a Boltzmann statistics to the barrier height difference of 0.11 eV, the probability to obtain a C-C, i.e., *pyridinic-*N, is 11%; which is in good agreement with the experimental value of 9.2%. It is important to note that the pyridyl orientation giving rise to the C-N bond is less favorable than the one for the C-C bond after deposition on Au(111) surface. For this reason, the majority of the pyridyl rings will initially have the wrong orientation and they will need to rotate in order to reach the preferred configuration for the C-N bond formation. As shown before, this rotation is indeed easily accessible during the different annealing steps (see Fig. SI 8 in the Supporting Information).

**Fig. 6:**
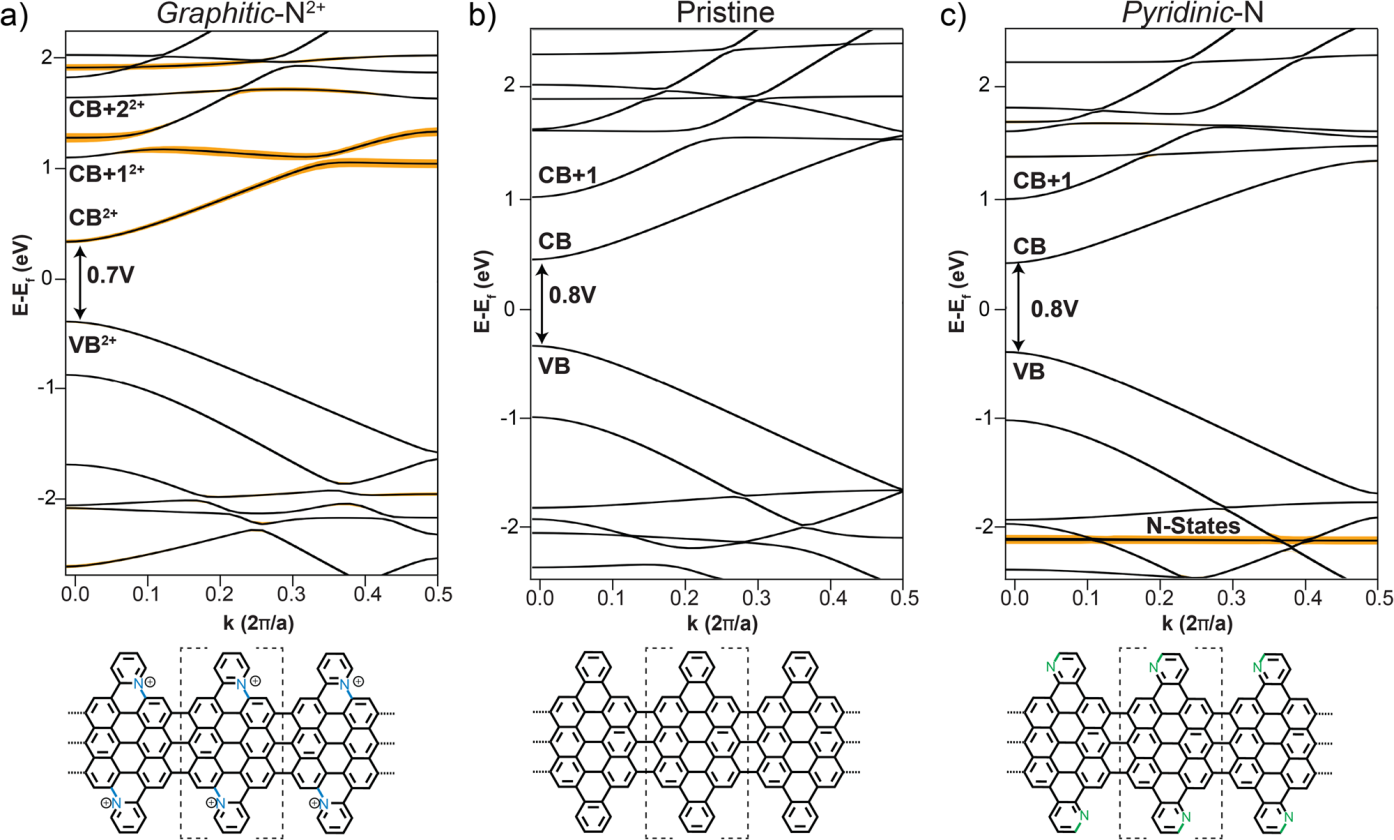
Gas-phase DFT band structures of possible edge-extended 7-AGNRs. Py-7-AGNR containing (**a**) *graphitic*-N^1+^ (**b**) pristine and (**c**) *pyridinic*-N. Bands with significant N contribution are highlighted by orange lines. The repetitive units are indicated by dotted square brackets in the chemical structure at the bottom of each panel. The g*raphitic*-N^1+^ case is modeled with one electron per unit cell removed. For the band structure of the charge-neutral case, see Fig. SI 13c.

The effect of N atoms on the electronic structures is investigated using DFT and compared to experimental results. The electronic contributions of *graphitic-*N atoms to the π-orbitals of the Py-7-AGNR on Au(111) were first investigated based on a Bader analysis^52^ (a schematic image showing the transfer of electron to the substrate is reported in Fig. SI 12); which, similar to other graphene nanoribbons containing N in this specific configuration^30,41^, shows a partial transfer of charges towards Au(111). In particular, we found that each *graphitic-*N loses 0.32 electron towards the substrate (i.e., 0.64 from each unit cell since it contains two *graphitic-*N atoms). Figure 6 and Fig. SI 13 show DFT band structures in gas phase of infinitively long Py-7-AGNR with the possible N configurations compared to the pristine one (partial density of states (PDOS) of the frontier bands are reported in SI 13). The *pyridinic-*N and the pristine GNRs share similar properties: the band gap in both cases is close to 0.8 eV. The effect of the *pyridinic-*N is the expected rigid downshift of valence band (VB) and conduction band (CB) by roughly 50 meV. The most significant contribution of *pyridinic-*N is a flat band at −2.1 eV (highlighted in yellow) which is mainly localized at the N positions. In contrast, *graphitic-*N has a stronger impact on the electronic properties. First, *graphitic-*N was considered as charge-neutral (Fig. SI 13c). Because of the extra electrons shared from the N atoms (one from each), the system starts to fill energy levels which are empty in the pristine case. In particular, the electrons populate the CB of the unsubstituted case, which now becomes the VB. This new ordering of the bands will shift E_f_ and it will give a small band gap of 50 meV. However, as shown at the beginning of the paragraph, the Py-7-AGNR containing *graphitic-*N is expected to lose part of its charge upon adsorption on Au(111); for this reason *graphitic-*N was also modeled removing one electron per unit cell, i.e., half-electron from each *graphitic-*N, as an approximation for charge transfer found from the Bader analysis. This allows to have a better comparison with experimental results. After removing the charge, E_f_ crosses the VB resulting in a partially filled band and in a metallic ribbon. In this way, the ordering of the orbitals is comparable to the charge neutral *graphitic-*N case (see Fig. SI 13c and SI 13 d): VB-1 is localized at the 7-AGNR sections and VB partially spreads over the pyridine extensions. Finally, CB is only localized close to the *graphitic-*N atoms and there are no analogous bands for the pristine case.

**Fig. 7:**
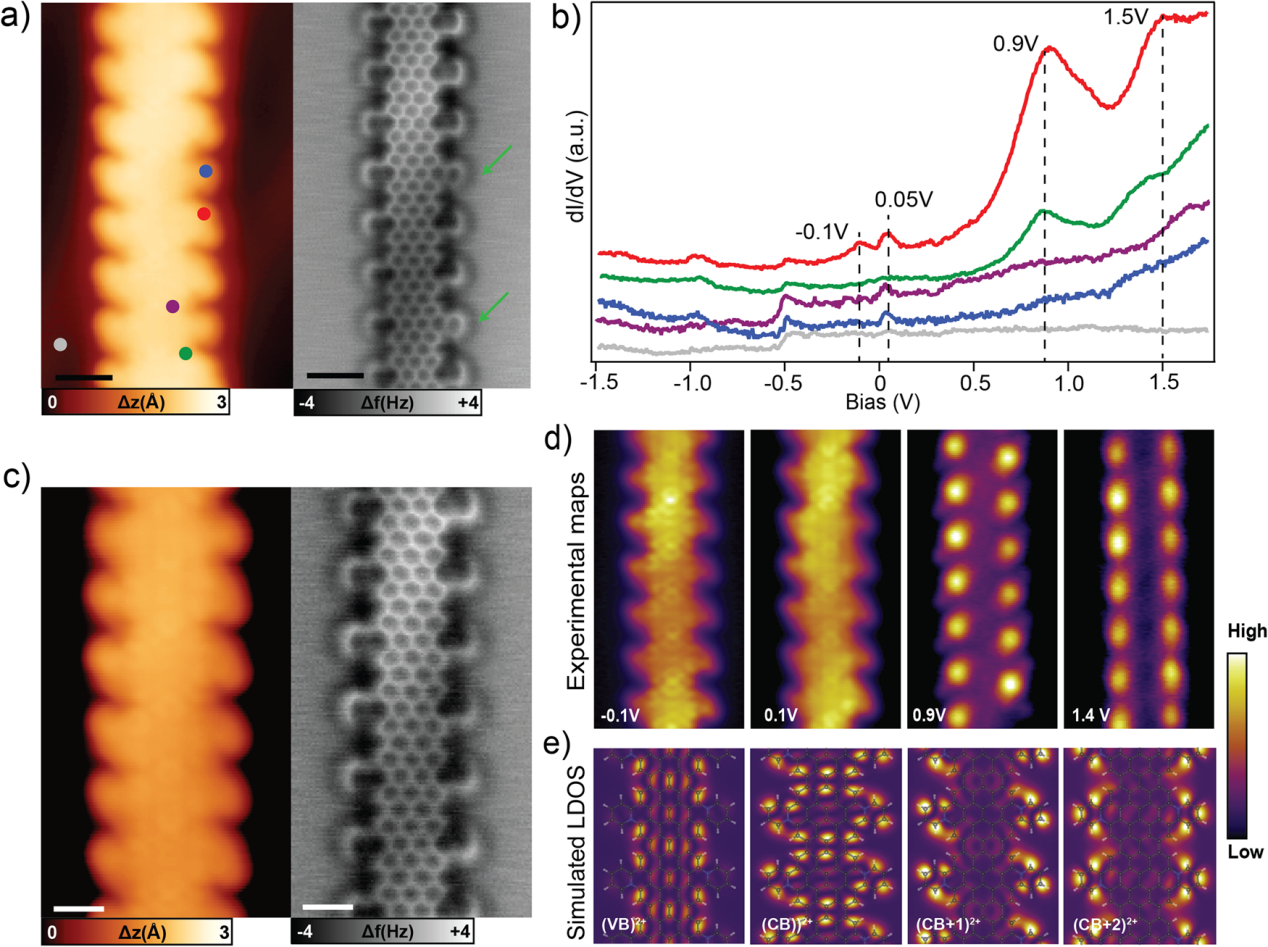
Electronic properties of Py-7-AGNRs. **a** STM (left) and nc-AFM (right) image of a Py-7-AGNR with *graphitic-*N edges. Two *pyridinic-*N defects are labeled by green arrows. (scale bar: 1 nm, scanning parameters:−1.5 V, 100 pA). **b** *dI/dV* spectra on different points (see **a**). (Open feedback parameters: −1.5 V, 200 pA; V_*rms*_: 20 mV) (**c**) STM (left) and nc-AFM (right) of another Py-7-AGNR segment without *pyridinic-*N defects. **d** *dI/dV* maps at selected bias voltages of the Py-7-AGNR shown in (**c**). **e** Simulated DOS maps of the singly charged system shown in Fig. 6a, evaluated at the different k-points of the corresponding bands.

Experimentally, we characterized the electronic properties of the Py-7-AGNR by means of STS. An example is reported in Fig. 7a. By analyzing the corresponding nc-AFM image, most of the edges present *graphitic-*N, except two on the right side (green arrow). The *dI/dV* spectra recorded at different locations are shown in Fig. 7b. The most prominent feature is an intense peak at 0.9 V, which is predominantly seen close to the N atoms of the pyridine extension (red and green spectra). Importantly, this contribution is only present at the *graphitic-*N edges. This is confirmed by taking a *dI/dV* spectrum close to a *pyridinic-*N (blue spectrum in Fig. 7b) which does not have any peak in this range, but is similar to the pristine GNR^48^. This agrees well with the gas-phase simulation shown in Fig. 6c, where the *pyridinic-*N states are localized far from the Fermi level (E_f_). Similar considerations can be made for the peak at 1.5 V, which is more intense only at the *graphitic-*N edges. These differences between the two distinct arrangements of N atoms point out that the electronic properties are affected by the specific position of the introduced heteroatom. There are two additional peaks close to E_f_ at −0.1 V and 0.05 V. To try to assign the peaks to the corresponding bands, we took *dI/dV* maps at selected bias voltages in order to map the distribution of these states in the ribbon, as shown in Fig. 7d, and compared them to simulated LDOS of a Py-7-AGNR with one electron per unit cell removed (Fig. 7e). The corresponding Py-7-AGNR is reported in panel c, where the nc-AFM image shows that the edges are all *graphitic-*N. The *dI/dV* maps recorded at −0.1 V reveals intensity spreading between the 7-AGNR backbone and the pyridine extensions. This resembles the map recorded above E_f_ (0.1 V) suggesting that it corresponds to the same band measured at different energies. Indeed, there is good agreement with the simulated LDOS maps of VB^1+^ measured at two different k-points. This confirms the non-integer charge transfer due to the *graphitic-*N atoms and the metallic behavior of the ribbon. The *dI/dV* map recorded at 0.9 V shows bright dots localized at the pyridine extensions closer to the N atoms. This behavior is well reproduced by the CB^1+^, which reproduces the inhomogeneous distribution at the edges. Finally, the state at 1.4 V is mainly localized at the pyridine extensions with a more symmetric distribution, in line with the simulation of CB + 1^1+^.

## Conclusion

In conclusion, we have reported the successful on-surface synthesis of long Py-7-AGNRs containing *graphitic-*N atoms at the edges. Their chemical structure has been elucidated by STM and nc-AFM techniques, revealing that the vast majority (more than 90%) of the pyridyl extensions form *graphitic-*N via C-N bond formation although the monomer design does not exclude *pyridinic-*N formation via C-C bond formation. The preferred C-N bond formation is rationalized by a lower energy barrier seen when approaching C and N and proves that subtle differences in reaction barriers allow to efficiently steer on-surface reactions. STS reveals a metallic ribbon for surface-adsorbed Py-7-AGNRs due to charge transfer to the substrate and a characteristic graphitic-N-related unoccupied state at 0.9 V. The results reported herein demonstrate the highly preferential formation of C-N bonds on metal surfaces by annealing, which contributes an important element to the design of new GNRs incorporating substitutional N atoms.

## Methods

Au(111) single crystal surfaces (MaTeck GmbH) were prepared by iterative Ar^+^ sputtering and annealing cycles. Prior to sublimation of molecules, the surface structure and cleanliness was checked by STM imaging. The molecular precursors DP-DPBA were filled into quartz crucibles of a home-built evaporator and sublimed at around 300 °C to achieve a rate of 0.45 Å min^-1^ onto the single crystal surfaces held at room temperature. STM and AFM measurements were performed with a commercial low-temperature STM/AFM from Scienta Omicron operated at a temperature of 4.5 K (LHe) and base pressure below 5 × 10^−11 ^mbar. STM images were acquired in constant-current mode (overview and high-resolution imaging), *dI/dV* spectra and *dI/dV* maps were acquired in constant-height mode. Differential conductance *dI/dV* spectra and maps were obtained with a lock-in amplifier. dI/dV parameters are reported in the figure captions. Bond-resolved nc-AFM images were acquired in constant-height mode with CO-functionalized tips at low bias voltages while recording frequency and current signals. Open feedback parameters and lowering of the tip height Δz are reported in the figure caption. The data were processed with Wavemetrics Igor Pro software.

DFT calculations were executed using the AiiDAlab^53^ applications, based on AiiDA^54^ workchains designed for the DFT code CP2K^55^ (systems adsorbed on gold) and for the DFT code Quantum Espresso^56^ (bandstructure calculations). Surface-adsorbate setups were modeled within a periodic slab scheme. The simulation cell included four Au atomic planes along the [111] orientation. Hydrogen atoms passivated one face of the slab to mitigate Au(111) surface states. A 40 Å vacuum layer was included to isolate the system from its periodic images along the axis orthogonal to the slab. Electronic wavefunctions were represented via TZV2P Gaussians basis sets for C, N, H, and DZVP for Au. Plane-waves basis set cutoff for the charge density was set at 600 Ry. Norm-conserving Goedecker–Teter–Hutter pseudopotentials were employed. The PBE GGA^57^ approximation for the exchange correlation functional was used and Grimme’s D3^58^ van der Waals corrections were included. Au supercells varied in size depending on the adsorbate, ranging from 28.12 × 26.54 Å^2^ (corresponding to 598 Au atoms) to 66.37 × 29.48 Å^2^ (1538 Au atoms). Geometry optimizations were performed with the bottom two atomic planes constrained while relaxing others until forces were below 0.005 eV ˚A. For nc-AFM simulations, DFT equilibrium geometries and electrostatic potentials were used alongside Hapala’s probe-particle code^59^.

For the bandstructure calculations, ultrasoft pseudopotentials, from the SSSP^60^ were employed to model the ionic potentials. A cutoff of 50 Ry (400 Ry) was used for the plane wave expansion of the wave functions (charge density). The simulation cell contained 15 Å of vacuum in the non-periodic directions to minimize interactions among periodic replicas of the system. The thickness of the vacuum region, the sampling of the BZ and the cutoff ensure convergency of the computed band structures. The atomic positions of the ribbon atoms and the cell dimension along the ribbon axis were optimized till forces were lower than 0.002 eV/A and the pressure in the cell was negligible. The band structures are aligned to the vacuum level computed from the average electrostatic potential in the vacuum region.

## Supplementary information

Supplementary Information

## Data Availability

The data that support the findings of this study are available in MaterialsClous with 10.24435/materialscloud:95-xw.

## References

[1] Cai, J. et al. Atomically precise bottom-up fabrication of graphene nanoribbons. *Nature* **466**, 470–473 (2010). 20651687 10.1038/nature09211

[2] Talirz, L. et al. On-surface synthesis and characterization of 9-atom wide armchair graphene nanoribbons. *ACS Nano* **11**, 1380–1388 (2017). 28129507 10.1021/acsnano.6b06405

[3] Yamaguchi, J. et al. Small bandgap in atomically precise 17-atom-wide armchair-edged graphene nanoribbons. *Commun. Mater.* **1**, 36 (2020).

[4] Kimouche, A. et al. Ultra-narrow metallic armchair graphene nanoribbons. *Nat. Commun.* **6**, 10177 (2015). 26658960 10.1038/ncomms10177 PMC4682157

[5] Chen, Y.-C. et al. Tuning the band gap of graphene nanoribbons synthesized from molecular precursors. *ACS Nano* **7**, 6123–6128 (2013). 23746141 10.1021/nn401948e

[6] Ruffieux, P. et al. On-surface synthesis of graphene nanoribbons with zigzag edge topology. *Nature* **531**, 489–492 (2016). 27008967 10.1038/nature17151

[7] Blackwell, R. E. et al. Spin splitting of dopant edge state in magnetic zigzag graphene nanoribbons. *Nature* **600**, 647–652 (2021). 34937899 10.1038/s41586-021-04201-y

[8] Teeter, J. D. et al. On- surface synthesis and spectroscopic characterization of laterally extended chevron graphene nanoribbons. *ChemPhysChem* **20**, 2281–2285 (2019). 31185134 10.1002/cphc.201900445

[9] Bronner, C. et al. Hierarchical on-surface synthesis of graphene nanoribbon heterojunctions. *ACS nano* **12**, 2193–2200 (2018). 29381853 10.1021/acsnano.7b08658

[10] Shekhirev, M., Zahl, P. & Sinitskii, A. Phenyl functionalization of atomically precise graphene nanoribbons for engineering interribbon interactions and graphene nanopores. *ACS nano* **12**, 8662–8669 (2018). 30085655 10.1021/acsnano.8b04489

[11] Moreno, C. et al. On-surface synthesis of superlattice arrays of ultra-long graphene nanoribbons. *Chem. Commun.* **54**, 9402–9405 (2018). 10.1039/c8cc04830d 30087965

[12] Son, Y.-W., Cohen, M. L. & Louie, S. G. Energy gaps in graphene nanoribbons. *Phys. Rev. Lett.* **97**, 216803 (2006). 17155765 10.1103/PhysRevLett.97.216803

[13] Houtsma, R. K., Rie, J. & Stöhr, M. Atomically precise graphene nanoribbons: interplay of structural and electronic properties. *Chem. Soc. Rev.* **50**, 6541–6568 (2021). 34100034 10.1039/d0cs01541e PMC8185524

[14] Yang, L., Park, C.-H., Son, Y.-W., Cohen, M. L. & Louie, S. G. Quasiparticle energies and band gaps in graphene nanoribbons. *Phys. Rev. Lett.* **99**, 186801 (2007). 17995426 10.1103/PhysRevLett.99.186801

[15] Durr, R. A. et al. Orbitally matched edge-doping in graphene nanoribbons. *J. Am. Chem. Soc.* **140**, 807–813 (2018). 29243927 10.1021/jacs.7b11886

[16] Nguyen, G. et al. Atomically precise graphene nanoribbon heterojunctions from a single molecular precursor. *Nat. Nanotechnol.* **12**, 1077–1082 (2017). 28945240 10.1038/nnano.2017.155

[17] Nguyen, G. D. et al. Bottom-up synthesis of n= 13 sulfur-doped graphene nanoribbons. *J. Phys. Chem. C.* **120**, 2684–2687 (2016).

[18] Zhang, Y.-F. et al. Sulfur-doped graphene nanoribbons with a sequence of distinct band gaps. *Nano Res.* **10**, 3377–3384 (2017).

[19] Ohtomo, M. et al. Effect of edge functionalization on the bottom-up synthesis of nanographenes. *ChemPhysChem* **20**, 3366–3372 (2019). 31596042 10.1002/cphc.201900510

[20] Cloke, R. R. et al. Site-specific substitutional boron doping of semiconducting armchair graphene nanoribbons. *J. Am. Chem. Soc.* **137**, 8872–8875 (2015). 26153349 10.1021/jacs.5b02523

[21] Kawai, S. et al. Atomically controlled substitutional boron-doping of graphene nanoribbons. *Nat. Commun.* **6**, 8098 (2015). 26302943 10.1038/ncomms9098 PMC4560828

[22] Bronner, C. et al. Aligning the band gap of graphene nanoribbons by monomer doping. *Angew. Chem. Int. Ed.* **52**, 4422–4425 (2013). 10.1002/anie.201209735 23512734

[23] Vo, T. H. et al. Bottom-up solution synthesis of narrow nitrogen-doped graphene nanoribbons. *Chem. Commun.* **50**, 4172–4174 (2014). 10.1039/c4cc00885e 24623056

[24] Gao, Y. et al. Selective activation of four quasi-equivalent C–H bonds yields n-doped graphene nanoribbons with partial corannulene motifs. *Nat. Commun.* **13**, 6146 (2022). 36253383 10.1038/s41467-022-33898-2 PMC9576682

[25] Zhang, Y. et al. On-surface synthesis of a nitrogen-doped graphene nanoribbon with multiple substitutional sites. *Angew. Chem. Int. Ed.* **61**, 202204736 (2022). 10.1002/anie.202204736 35452167

[26] Carbonell-Sanroma, E. et al. Doping of graphene nanoribbons via functional group edge modification. *ACS Nano* **11**, 7355–7361 (2017). 28636331 10.1021/acsnano.7b03522

[27] Li, J. et al. Band depopulation of graphene nanoribbons induced by chemical gating with amino groups. *ACS Nano* **14**, 1895–1901 (2020). 31999431 10.1021/acsnano.9b08162

[28] Pawlak, R. et al. Bottom-up synthesis of nitrogen-doped porous graphene nanoribbons. *J. Am. Chem. Soc.* **142**, 12568–12573 (2020). 32589029 10.1021/jacs.0c03946

[29] Zhang, Y. et al. Direct visualization of atomically precise nitrogen-doped graphene nanoribbons. *Appl. Phys. Lett.* **105**, 023101 (2014).

[30] Wen, E. C. H. et al. Fermi-level engineering of nitrogen core-doped armchair graphene nanoribbons. *J. Am. Chem. Soc.* **145**, 19338–19346 (2023). 37611208 10.1021/jacs.3c05755 PMC10485924

[31] Maaß, F. et al. Electronic structure changes during the on-surface synthesis of nitrogen-doped chevron-shaped graphene nanoribbons. *Phys. Rev. B* **96**, 045434 (2017).

[32] Vo, T. H. et al. Nitrogen-Doping induced self-assembly of graphene nanoribbon-based two-dimensional and three-dimensional metamaterials. *Nano Lett.* **15**, 5770–5777 (2015). 26258628 10.1021/acs.nanolett.5b01723

[33] Eimre, K. et al. On-surface synthesis and characterization of nitrogen-substituted undecacenes. *Nat. Commun.* **13**, 511 (2022). 35082284 10.1038/s41467-022-27961-1 PMC8791976

[34] Kawai, S. et al. Multiple heteroatom substitution to graphene nanoribbon. *Sci. Adv.* **4**, 7181 (2018). 10.1126/sciadv.aar7181 PMC5898832 29662955

[35] Lazar, P., Mach, R. & Otyepka, M. Spectroscopic fingerprints of graphitic, pyrrolic, pyridinic, and chemisorbed nitrogen in n-doped graphene. *J. Phys. C.* **123**, 10695–10702 (2019).

[36] Yamada, Y., Tanaka, H., Kubo, S. & Sato, S. Unveiling bonding states and roles of edges in nitrogen-doped graphene nanoribbon by x-ray photoelectron spectroscopy. *Carbon* **185**, 342–367 (2021).

[37] Costa Azevedo, A. S., Saraiva-Souza, A., Meunier, V. & Girao, E. C. Electronic properties of n-rich graphene nanochevrons. *Phys. Chem. Chem. Phys.* **23**, 13204–13215 (2021). 34085086 10.1039/d1cp00197c

[38] Cai, J. et al. Graphene nanoribbon heterojunctions. *Nat. Nanotech.* **9**, 896–900 (2014). 10.1038/nnano.2014.184 25194948

[39] Basagni, A. et al. Tunable band alignment with unperturbed carrier mobility of on-surface synthesized organic semiconducting wires. *ACS Nano* **10**, 2644–2651 (2016). 26841052 10.1021/acsnano.5b07683 PMC4783043

[40] Lv, Y. et al. Activating impurity effect in edge nitrogen-doped chevron graphene nanoribbons. *J. Phys. Commun.* **2**, 045028 (2018).

[41] Wen, E. C. H. et al. Magnetic interactions in substitutional core doped graphene nanoribbons. *J. Am. Chem. Soc.* **144**, 13696–13703 (2022). 35867847 10.1021/jacs.2c04432

[42] Talirz, L., Ruffieux, P. & Fasel, R. On-surface synthesis of atomically precise graphene nanoribbons. *Adv. Mater.* **28**, 6222–6231 (2016). 26867990 10.1002/adma.201505738

[43] Sun, K. et al. Heterocyclic ring-opening of nanographene on Au(111). *Angew. Chem.* **133**, 9513–9518 (2021). 10.1002/anie.202017137 33576120

[44] Piskun, I. et al. Covalent C–N bond formation through a surface catalyzed thermal cyclodehydrogenation. *J. Am. Chem. Soc.* **142**, 3696–3700 (2020). 32043869 10.1021/jacs.9b13507

[45] Xu, X. et al. On-surface activation of benzylic C-H bonds for the synthesis of pentagon-fused graphene nanoribbons. *Nano Res.* **14**, 4754–4759 (2021).

[46] Tenorio, M. et al. Introducing design strategies to preserve N-heterocycles throughout the on-surface synthesis of graphene nanostructures. *Small Methods* **8**, 2300768 (2024). 10.1002/smtd.202300768 37840449

[47] Moreno, C. et al. Molecular bridge engineering for tuning quantum electronic transport and anisotropy in nanoporous graphene. *J. Am. Chem. Soc.* **145**, 8988–8995 (2023). 36988648 10.1021/jacs.3c00173 PMC10141406

[48] Moreno, C. et al. Bottom-up synthesis of multifunctional nanoporous graphene. *Science* **360**, 199–203 (2018). 29650671 10.1126/science.aar2009

[49] Gross, L., Mohn, F., Moll, N., Liljeroth, P. & Meyer, G. The chemical structure of a molecule resolved by atomic force microscopy. *Science* **325**, 1110–1114 (2009). 19713523 10.1126/science.1176210

[50] Biswas, K. et al. On-surface synthesis of a dicationic diazahexabenzocoronene derivative on the Au(111) surface. *Angew. Chem. Int. Ed.* **60**, 25551–25556 (2021). 10.1002/anie.202111863 PMC9298296 34546628

[51] Wang, X.-Y. et al. Exploration of pyrazine-embedded antiaromatic polycyclic hydrocarbons generated by solution and on-surface azomethine ylide homocoupling. *Nat. Comm.* **8**, 1948 (2017). 10.1038/s41467-017-01934-1 PMC5717246 29208962

[52] Kumar, P. S. V., Raghavendra, V. & Subramanian, V. Bader’s theory of atoms in molecules (aim) and its applications to chemical bonding. *J. Chem. Sci.* **128**, 1527–1536 (2016).

[53] Yakutovich, A. V. et al. Aiidalab an ecosystem for developing, executing, and sharing scientific workflows. *Comput. Mater. Sci.* **188**, 110165 (2021).

[54] Pizzi, G., Cepellotti, A., Sabatini, R., Marzari, N. & Kozinsky, B. Aiida: automated interactive infrastructure and database for computational science. *Comput. Mater. Sci.* **111**, 218–230 (2016).

[55] Hutter, J., Iannuzzi, M., Schiffmann, F. & VandeVondele, J. cp2k: atomistic simulations of condensed matter systems.*Wiley Interdiscip. Rev. Comput. Mol. Sci.* **4**, 15–25 (2014).

[56] Giannozzi, P. et al. Quantum espresso: a modular and open-source software project for quantum simulations of materials. *J. Phys. Condens Matter* **21**, 395502 (2009). 21832390 10.1088/0953-8984/21/39/395502

[57] Perdew, J. P., Burke, K. & Ernzerhof, M. Generalized gradient approximation made simple. *Phys. Rev. B* **54**, 16533 (1996). 10.1103/PhysRevLett.77.3865 10062328

[58] Grimme, S., Antony, J., Ehrlich, S. & Krieg, H. A consistent and accurate ab initio parametrization of density functional dispersion correction (DFT-D) for the 94 elements H-Pu. *J. Chem. Phys.* **132**, 154104 (2010). 20423165 10.1063/1.3382344

[59] Hapala, P. et al. Mechanism of high-resolution STM/AFM imaging with functionalized tips. *Phys. Rev. B* **90**, 085421 (2014).

[60] Probert, M. I. J. et al. Reproducibility in density functional theory calculations of solids. *Science* **351**, 3000 (2016). 10.1126/science.aad3000 27013736

