# Supplementary material for: Preferential graphitic-nitrogen formation in pyridine-extended graphene nanoribbons
Source: Commun Chem. 2024 Nov 21;7:274. doi: 10.1038/s42004-024-01344-7 (PMC11582605; doi:10.1038/s42004-024-01344-7)
Supplement: Supplementary file 1 — Supplementary Information [file 42004_2024_1344_MOESM1_ESM.pdf]

Preferential Graphitic-Nitrogen Formation in Pyridine-Extended Graphene Nanoribbons

SUPPORTING INFORMATION

Nicolò Bassi[1†], Xiushang Xu[2†], Feifei Xiang[1], Nils Krane[1], Carlo A. Pignedoli[1], Akimitsu Narita[2*], Roman Fasel[1,3], Pascal Ruffieux[1*]

[1] nanotech@surfaces Laboratory, Empa, Swiss Federal Laboratories for Materials Science and Technology, 8600 Dübendorf, Switzerland.

[2] Organic and Carbon Nanomaterials Unit, Okinawa Institute of Science and Technology Graduate University, Okinawa, Japan.

[3] Department of Chemistry, Biochemistry and Pharmaceutical Sciences, University of Bern, 3012 Bern, Switzerland.

* Corresponding author(s). E-mail(s): pascal.ruffieux@empa.ch; akimitsu.narita@oist.jp

† These authors contributed equally to this work.

Keywords: Graphene nanoribbons, *Graphitic*-N, Nitrogen substitution, On-surface synthesis, scanning tunneling microscopy

Synthetic procedures

All reactions working with air- or moisture-sensitive compounds were carried out under argon atmosphere using standard Schlenk line techniques. Unless otherwise noted, all starting materials and other chemicals were purchased from commercial sources and used without further purification. 9,9'-Bianthracene-2,2'-diyl bis(trifluoromethanesulfonate) (**2**) was prepared according to our previously reported procedure[1].Thin-layer chromatography (TLC) was done on silica gel coated aluminum sheets with F254 indicator and column chromatography separation was performed with silica gel (particle size 0.063-0.200 mm). Nuclear Magnetic Resonance (NMR) spectra were recorded in CD_2Cl_2 using Bruker AVANCE NEO 500 MHz NMR spectrometer. Chemical shifts (*δ*) were expressed in ppm relative to the residual solvents (CD_2Cl_2, ^{1}H: 5.32 ppm, ^{13}C: 54.00 ppm; $CDCl_3$, ^{1}H: 7.26 ppm, ^{13}C: 76.00 ppm). Coupling constants (*J*) were recorded in Hertz. Abbreviations: s = singlet, d = doublet, t = triplet, q = quartet, m = multiplet. High-resolution mass spectra (HRMS) were recorded on Thermo Scientific LTQ-Orbitrap Mass Spectrometer by electrospray ionization (ESI).

2,2'-bis(4,4,5,5-tetramethyl-1,3,2-dioxaborolan-2-yl)-9,9'-bianthracene （**3**）

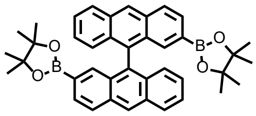

A 50-mL Schlenk tube was charged with 9,9'-bianthracene-2,2'-diyl bis(trifluoromethanesulfonate) (**2**) (400 mg, 0.615 mmol), bis(pinacolato)diboron (467 mg, 1.84 mmol), potassium acetate (KOAc) (270 mg, 3.00 mmol), [1,1′-bis(diphenylphosphino)ferrcene]dichloropalladium(II) (Pd(dppf)Cl_2) (52.0 mg, 61.5 µmol), and anhydrous dimethylformamide (DMF) (6.0 mL). After degassing by argon bubbling for 15 min, the reaction mixture was heated at 85 °C under argon atmosphere for 16 h. After cooling to a room temperature, the resulting mixture was poured into water (50 mL) and extracted with diethyl ether (10 mL) for three times. The organic phases were combined, washed with brine, dried over $MgSO_4$, and evaporated. The residue was purified by silica gel column chromatography to give the title compound as light yellow solid (281 mg, 75% yield. ^{1}H NMR (500 MHz, $CDCl_3$) *δ* 8.66 (s, 2H), 8.15 – 8.13 (m, 4H), 7.81 (d, *J* = 8.5 Hz, 2H), 7.67 (d, *J* = 1.3 Hz, 2H), 7.44 – 7.35 (m, 2H), 7.08 – 7.02 (m, 2H), 6.97 – 6.81 (m, 2H), 1.16 (d, *J* = 2.4 Hz, 24H). ^{13}C NMR (126 MHz, $CDCl_3$) δ 134.43, 133.26, 131.62, 131.16, 130.93, 130.37, 128.42, 127.34, 126.54, 126.26, 126.19, 124.55, 82.63, 23.67.HRMS (ESI, Positive): *m/z* Calcd. For $C_{40}H_{41}B_2O_4^+$: 607.3185 [M+H]$^+$, found:607.3161.

2,2'-di(pyridin-2-yl)-9,9'-bianthracene (**4**)

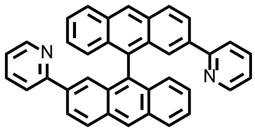

A 30-mL Schlenk tube was charged with compound **3** (30 mg, 49 µmol), 2-bromopyridine (39 mg, 0.25 mmol), Pd(PPh_3)$_4$ (5.7 mg, 4.9 µmol), K_2CO_3 (2.0 M aqueous solution, 0.5 mL), and tetrahydrofuran (THF) (2.0 mL). After degassing by argon bubbling for 10 min, the reaction mixture was heated at 80 °C under argon atmosphere for 20 h. The resulting mixture was cooled to a room temperature, and then poured into water (20 mL), followed by extraction with CH_2Cl_2 (10 mL) for three times. The organic phases were combined, washed with brine, dried over $MgSO_4$, and evaporated. The residue was purified by silica gel column chromatography to give the title compound as light yellow solid (16 mg, 66% yield). ^{1}H NMR (500 MHz, CD_2Cl_2) *δ* 8.70 (s, 2H), 8.38 – 8.36 (m, 2H), 8.24 (d, *J* = 9.0 Hz, 2H), 8.16 – 8.08 (m, 4H), 7.64 (dd, *J* = 1.7, 0.9 Hz, 2H), 7.42 – 7.39 (m, 4H), 7.24 – 7.19 (m, 2H), 7.12 – 7.03 (m, 2H), 7.00 – 6.91 (m, 4H). ^{13}C NMR (126 MHz, CD_2Cl_2) *δ* 156.79, 149.46, 136.90, 136.48, 133.82, 132.08, 131.67, 131.56,

129.30, 128.71, 127.37, 126.62, 126.13, 125.68, 124.54, 122.03, 120.72. HRMS (ESI, Positive): *m/z* Calcd. For $C_{38}H_{25}N_2^+$: 509.2012 $[M+H]^+$, found: 509.1996.

10,10'-dibromo-2,2'-di(pyridin-2-yl)-9,9'-bianthracene (**1**)

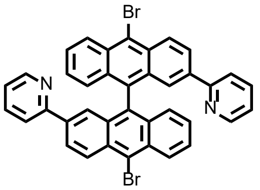

A 50-mL Schlenk tube was charged with compound **4** (120 mg, 0.236 mmol), *N*-bromosuccinimide (NBS) (168 mg, 0.945 mmol), and CH_2Cl_2 (10 mL). The resulting mixture was stirred at room temperature overnight. After quenching with acetone (10 mL), the solvents were evaporated and the residue was purified by silica gel column chromatography to the tittle compound **1** as light yellow solid (141 mg, 90% yield). ^1H NMR (500 MHz, CD_2Cl_2) δ 8.76 (d, J = 9.3 Hz, 2H), 8.67 – 8.64 (m, 2H), 8.42 – 8.39 (m, 2H), 8.26 (dd, J = 9.3, 1.8 Hz, 2H), 7.66 (d, J = 1.7 Hz, 2H), 7.55 – 7.53 (m, 2H), 7.44 – 7.42 (m, 2H), 7.23 – 7.19 (m, 2H), 7.14 – 7.10 (m, 2H), 7.02-6.98 (m, 2H), 6.98 – 6.95 (m, 2H). ^{13}C NMR (126 MHz, CD_2Cl_2) δ 173.25, 155.98, 149.57, 137.36, 136.61, 133.94, 132.67, 132.34, 130.90, 130.44, 128.80, 128.15, 127.59, 127.07, 126.64, 126.45, 124.70, 124.03, 122.39, 120.91. HRMS (ESI, Positive): *m/z* Calcd. For $C_{38}H_{23}Br_2N_2^+$: 665.0223 $[M+H]^+$, found:665.0209.

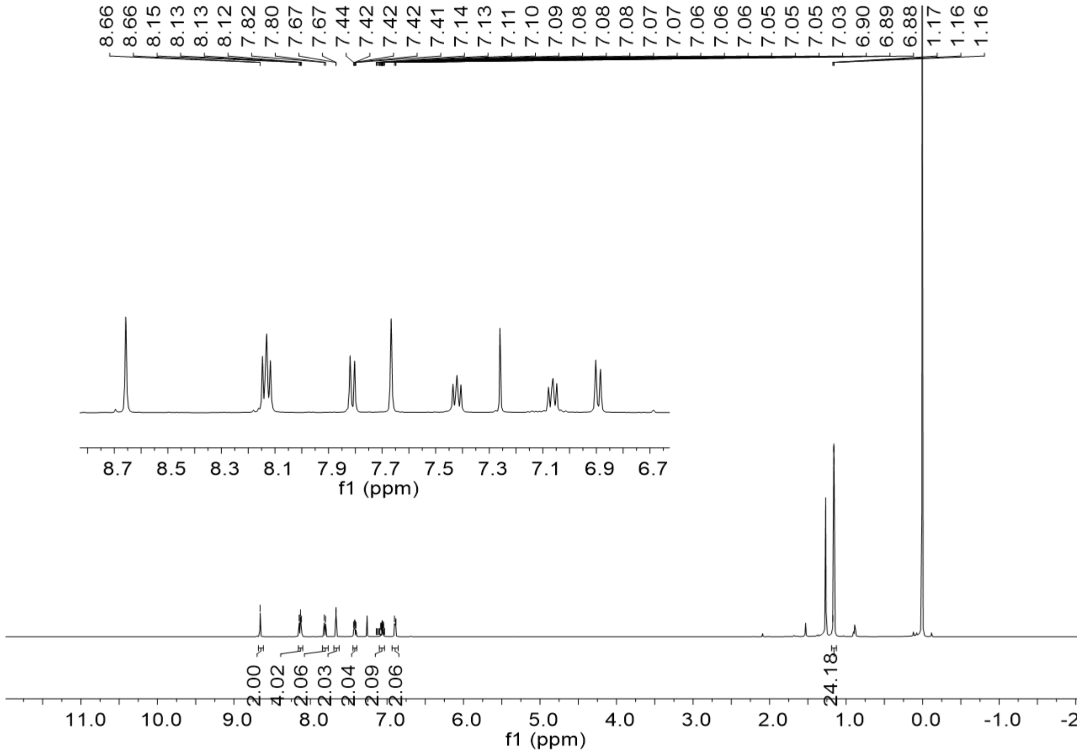

Figure SI 1. ^1H NMR spectrum of compound **3** in $CDCl_3$ (500 MHz, 298 K).

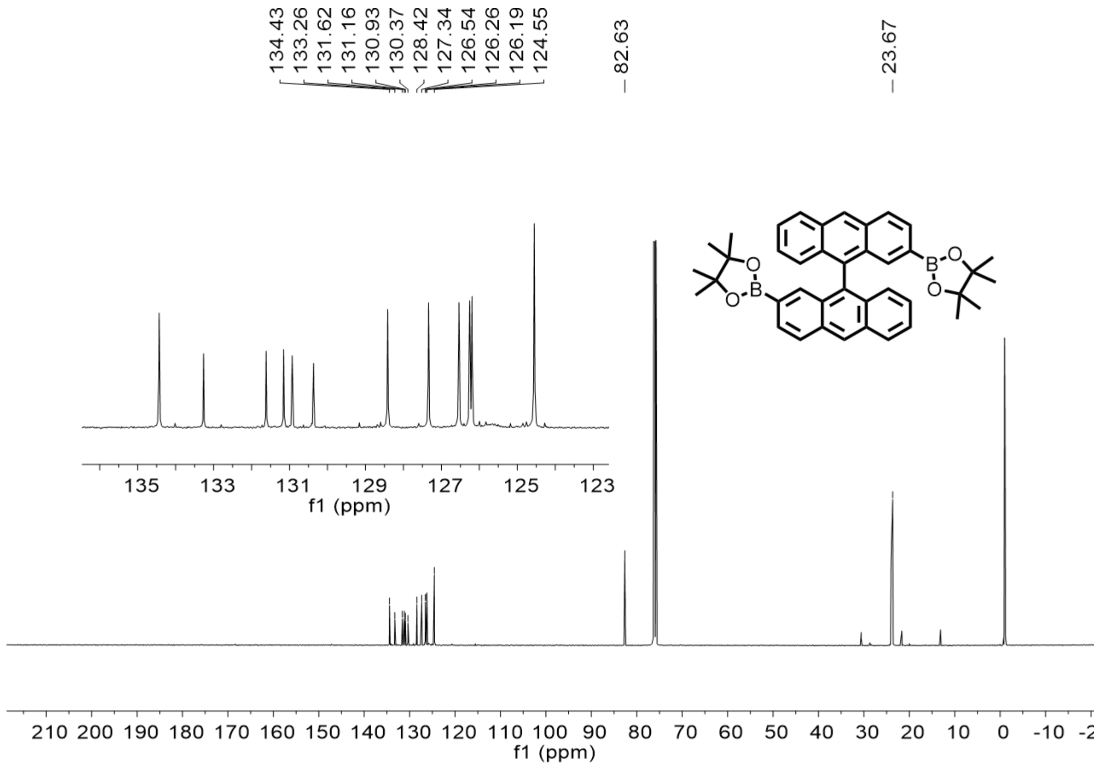

Figure SI 2. ^{13}C NMR spectrum of compound **3** in $CDCl_3$ (126 MHz, 298 K).

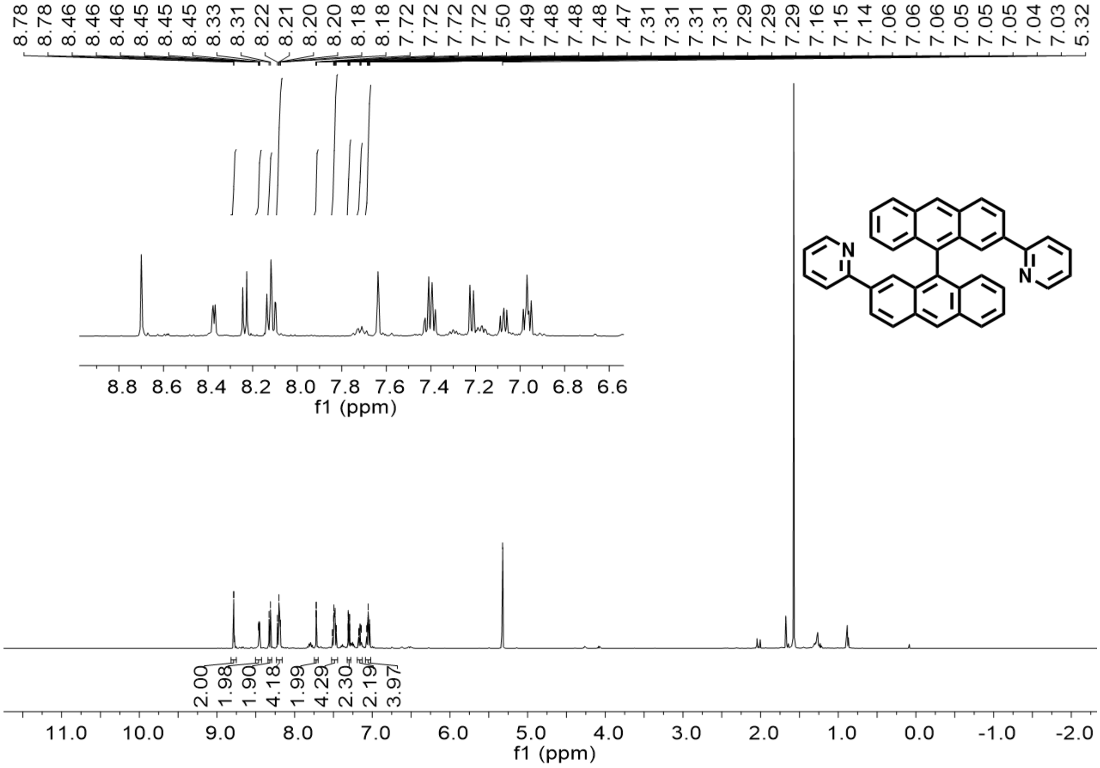

Figure SI 3. ^{1}H NMR spectrum of compound **4** in CD_2Cl_2 (500 MHz, 298 K).

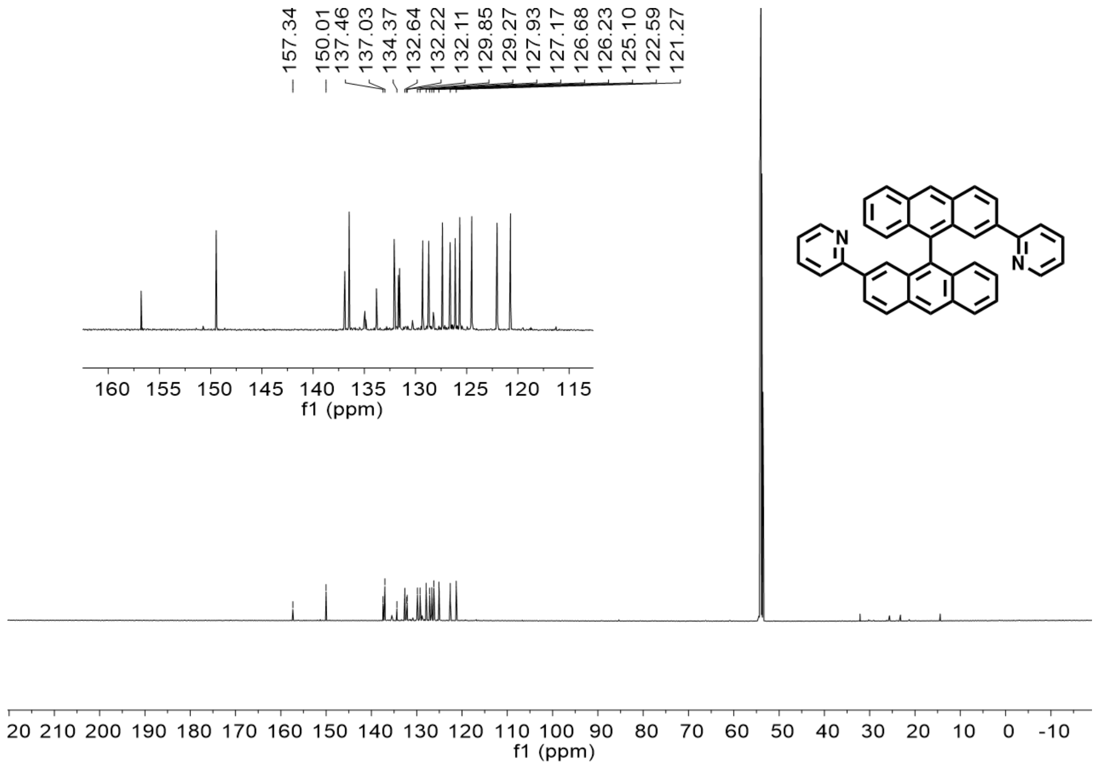

Figure SI 4. ^{13}C NMR spectrum of compound **4** in CD_2Cl_2 (126 MHz, 298 K).

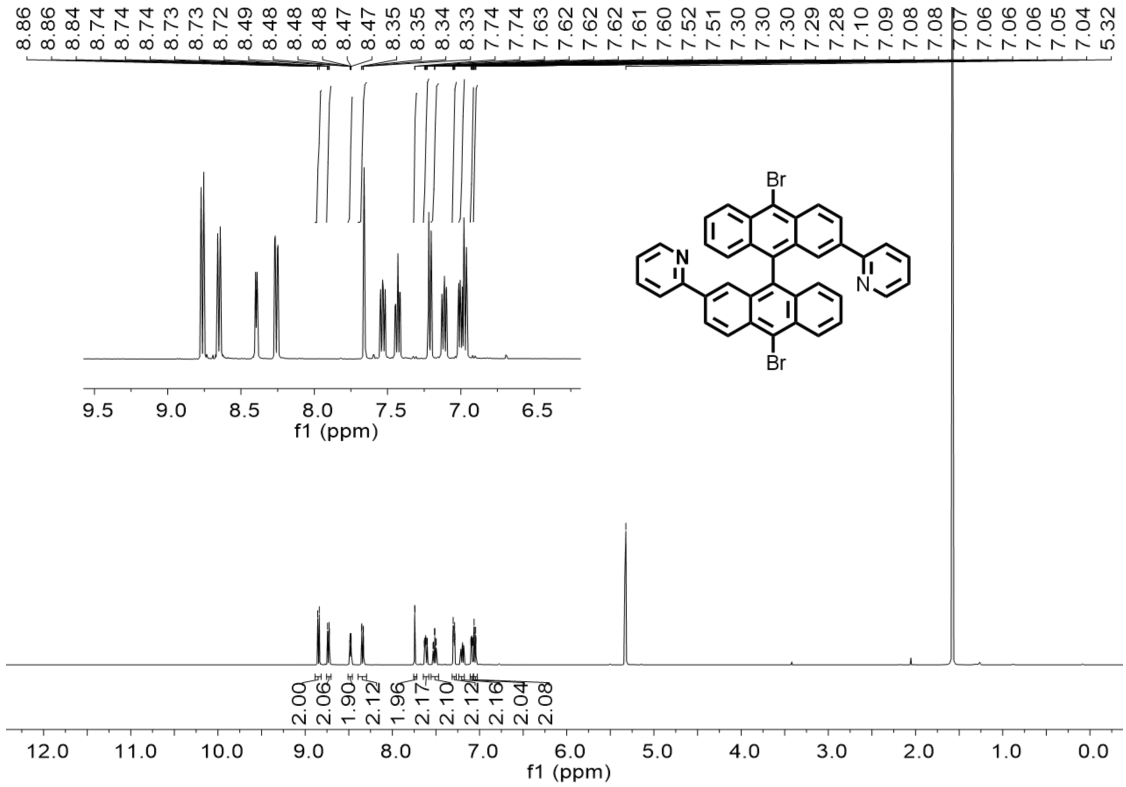

Figure SI 5. ^{1}H NMR spectrum of compound **1** in CD_2Cl_2 (500 MHz, 298 K).

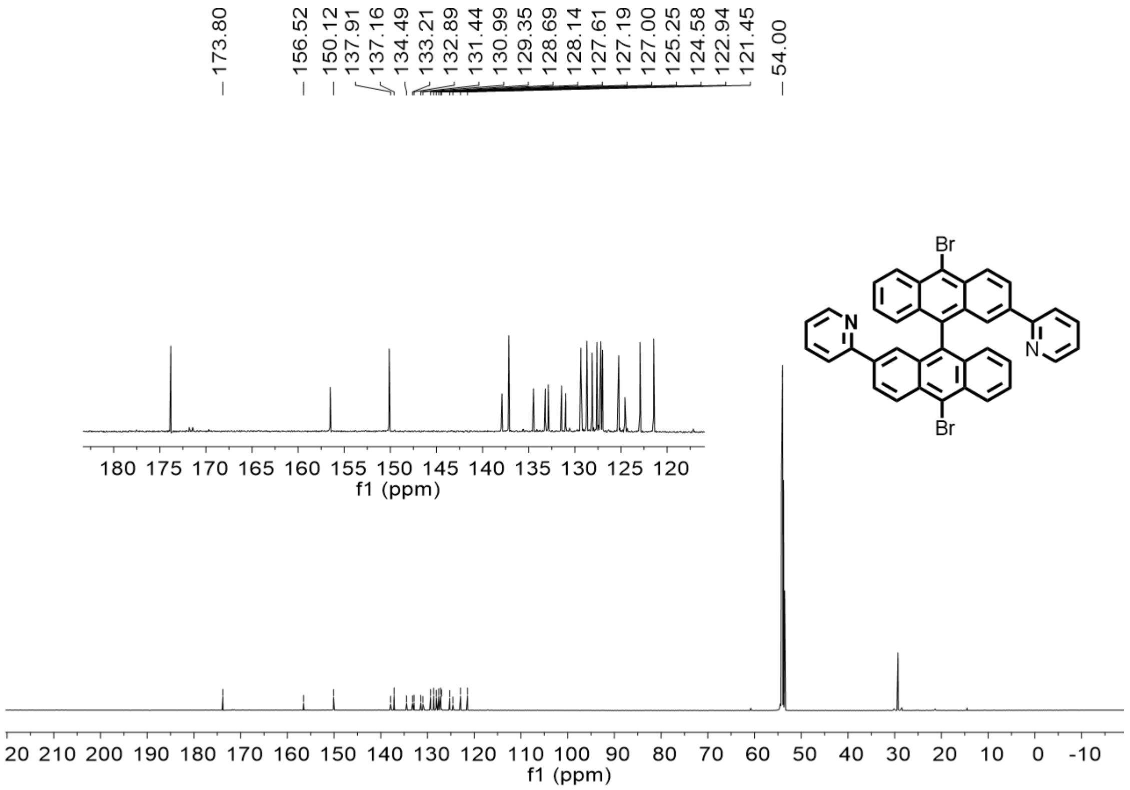

Figure SI 6. ^{13}C NMR spectrum of compound **1** in CD_2Cl_2 (126 MHz, 298 K).

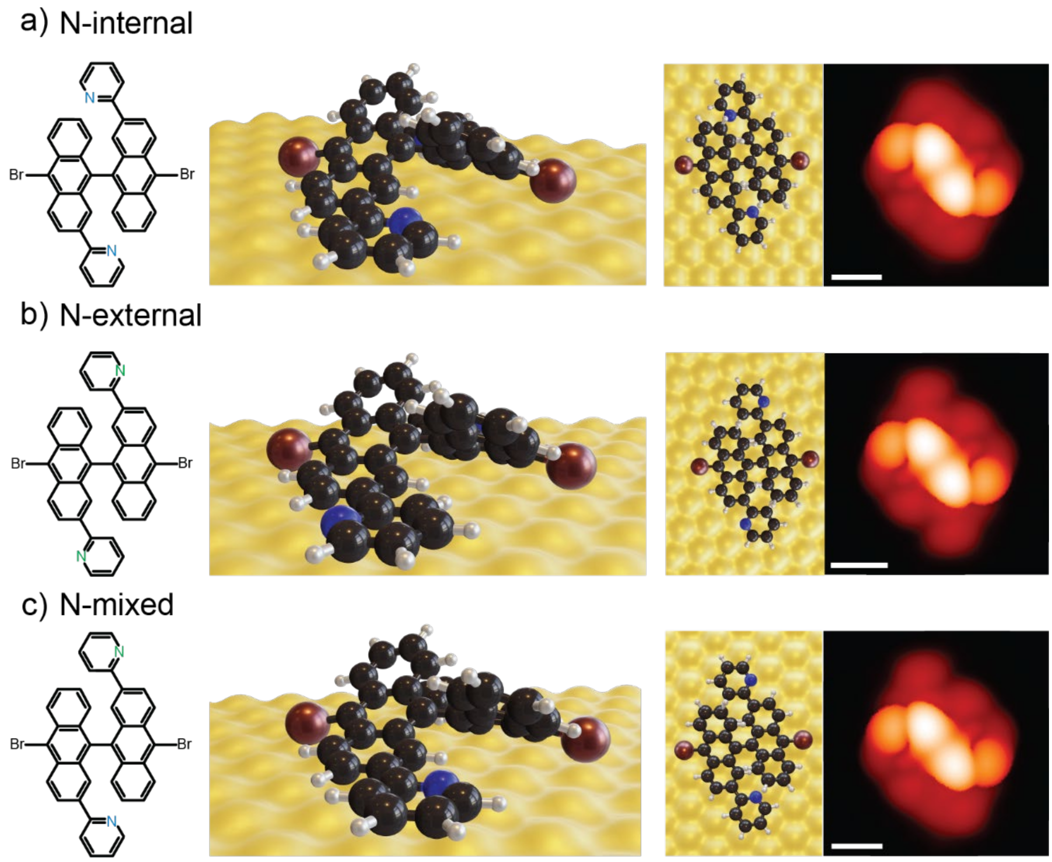

Figure SI 7: DFT-relaxed geometries of DB-DPBA with different pyridyl orientations. a) N atoms pointing towards the bianthracene unit (from left: chemical structure of the pyridyl orientation, DFT-relaxed structure (perspective view), DFT-relaxed structure (top view) and corresponding STM simulation). b) N atoms pointing away from the bianthracene unit (from left: chemical structure of the pyridyl orientation, DFT-relaxed structure (perspective view), DFT-relaxed structure (top view) and corresponding STM simulation). c) N atoms pointing in both directions with respect to the bianthracene unit (from left: chemical structure of the pyridyl orientation, DFT-relaxed structure (perspective view), DFT-relaxed structure (top view) and corresponding STM simulation). The three different configurations are quite similar and difficult to distinguish from STM. Scale bars: 1 nm

Table 1: Structural properties of the three different pyridyl orientations

	N-internal	N-external	N-mixed
Maximum height (nm)	0.663	0.651	0.665
Distance bright dots (nm)	0.793	0.795	0.795
Energy (eV)	-443812.192	-443812.305	-443812.265
ΔE	0.113	0	0.04

Table summarizing structural parameters of the three different geometries after optimization. DFT relaxed structure are reported in Figure SI 7

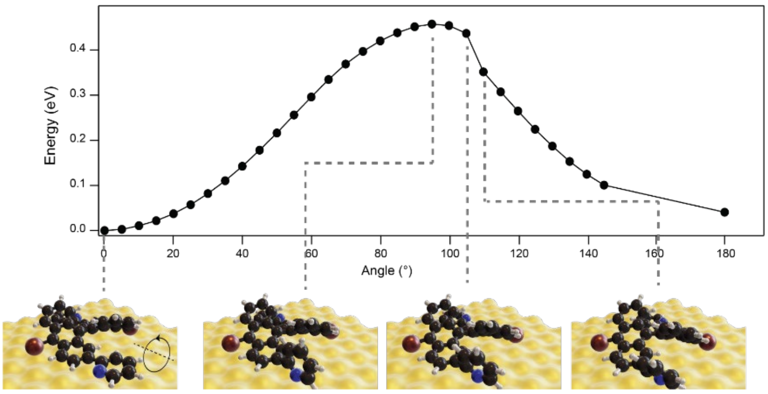

Figure SI 8: Energy barrier for rotation. Energy barrier to flip the pyridyl ring of 180°. The x- axis refers to the dihedral angle of the pyridyl unit (rotation direction reported in the left panel). The energy barrier needed to overcome is 0.45 eV at an angle of 95°. Unconstrained geometry optimization of the structure corresponding to 145° leads to the local minimum corresponding to 180°. Bottom row: DFT-optimized geometry (perspective view) at significant points. The geometry after complete rotation corresponds to figure SI 7C

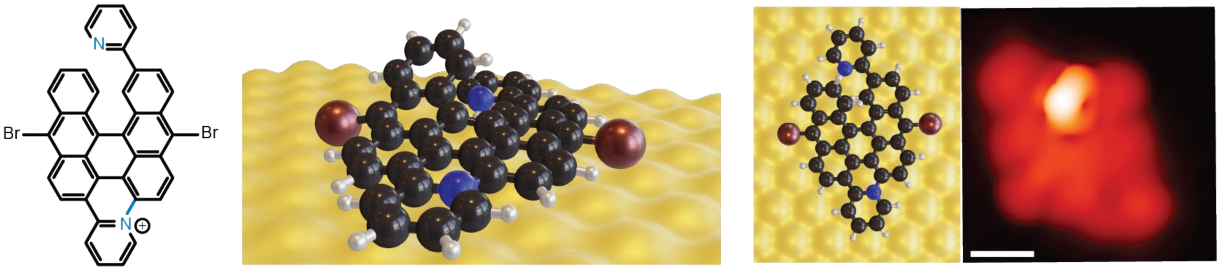

Figure SI 9: Partially planar structure. Partially planar structure after annealing at 250 °C. These agree with our experimental evidences of the sample. From left: chemical structure of the pyridyl orientation, equilibrium geometry obtained by DFT (perspective view), DFT-relaxed structure (top view) and corresponding STM simulation.

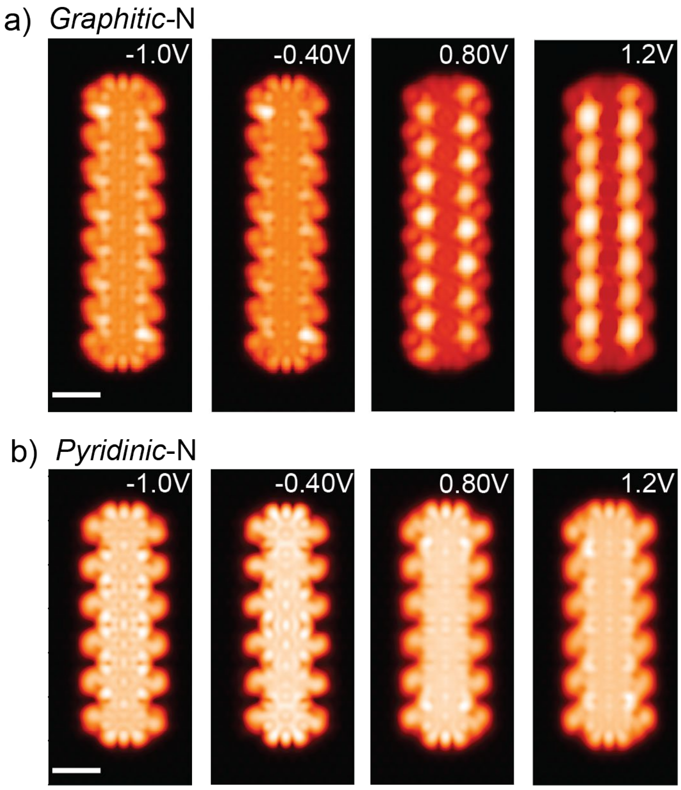

Figure SI 10: STM simulations at selected voltages. STM simulations based on DFT relaxed structures of Py-7-AGNR containing only a) *graphitic*-N and b) *pyridinic*-N. The *graphitic*-N edges appear bent at negative bias (upward on the left edge while downward on the right) and at positive values, there are high intensity states localized at the N positions. The *pyridinic*-N edges appear, instead, more rounded and symmetric without any signal at positive bias. These simulations match well with our experimental images. Scale bar: 1 nm. The simulated voltages are reported in the inset of each panel.

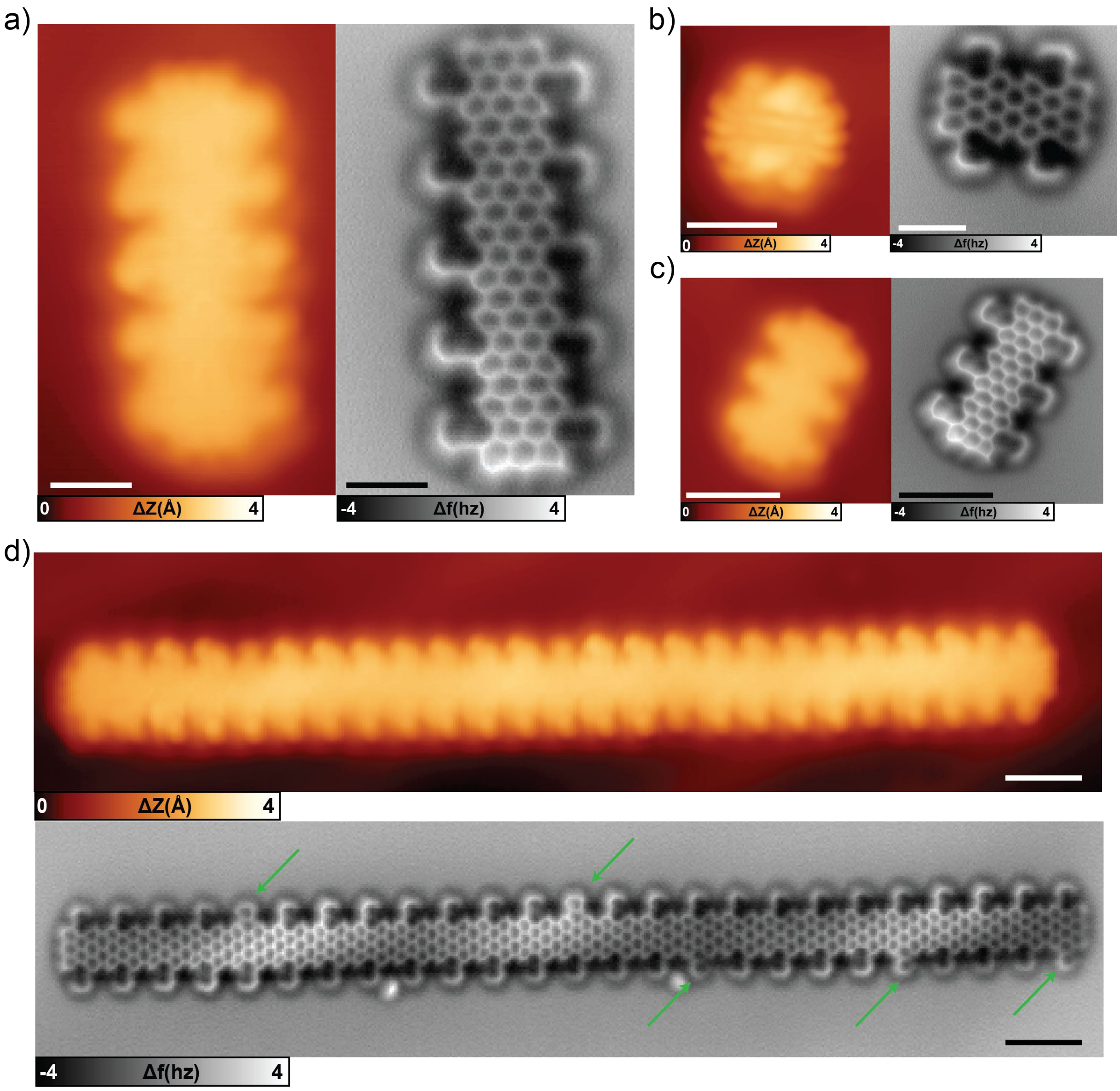

Figure SI 11: STM and nc-AFM images of different nanoribbons. a) pentamer (scale bar 1 nm, scanning parameter:-0.1 V, 100 pA. b) dimer (scale bar 1 nm, scanning parameter:- 0.1 V, 100 pA c) trimer (scale bar 2 nm, scanning parameter:-0.1 V, 100 pA). d) long ribbon (scale bar 2 nm, scanning parameter:-0.7 V, 70 pA). It can be noticed that most of the edge extensions exhibit graphitic-N, except the ones highlighted by green arrows in panel d.

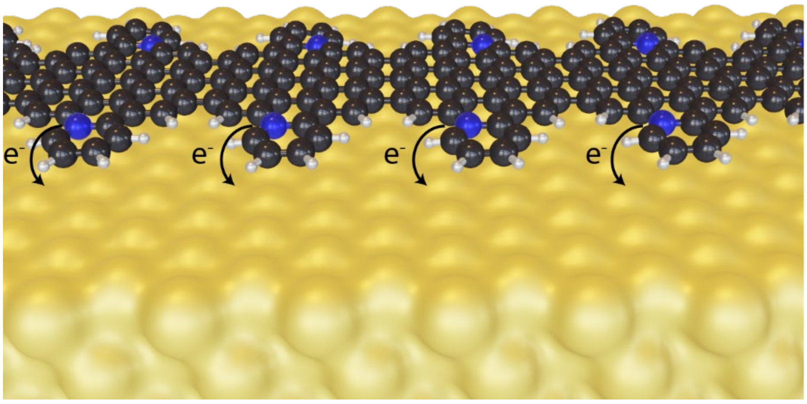

Figure SI 12: Schematic representation of the charge transfer to the substrate. Schematic representation of the charge transfer resulting from Bader analysis of a 6-units long Py-7-AGNR with *graphitic*-N on Au(111). The charge analysis reveal a transfer of 0.32 electron transfer *per* N-atom (i.e. 0.64 *per* unit cell).

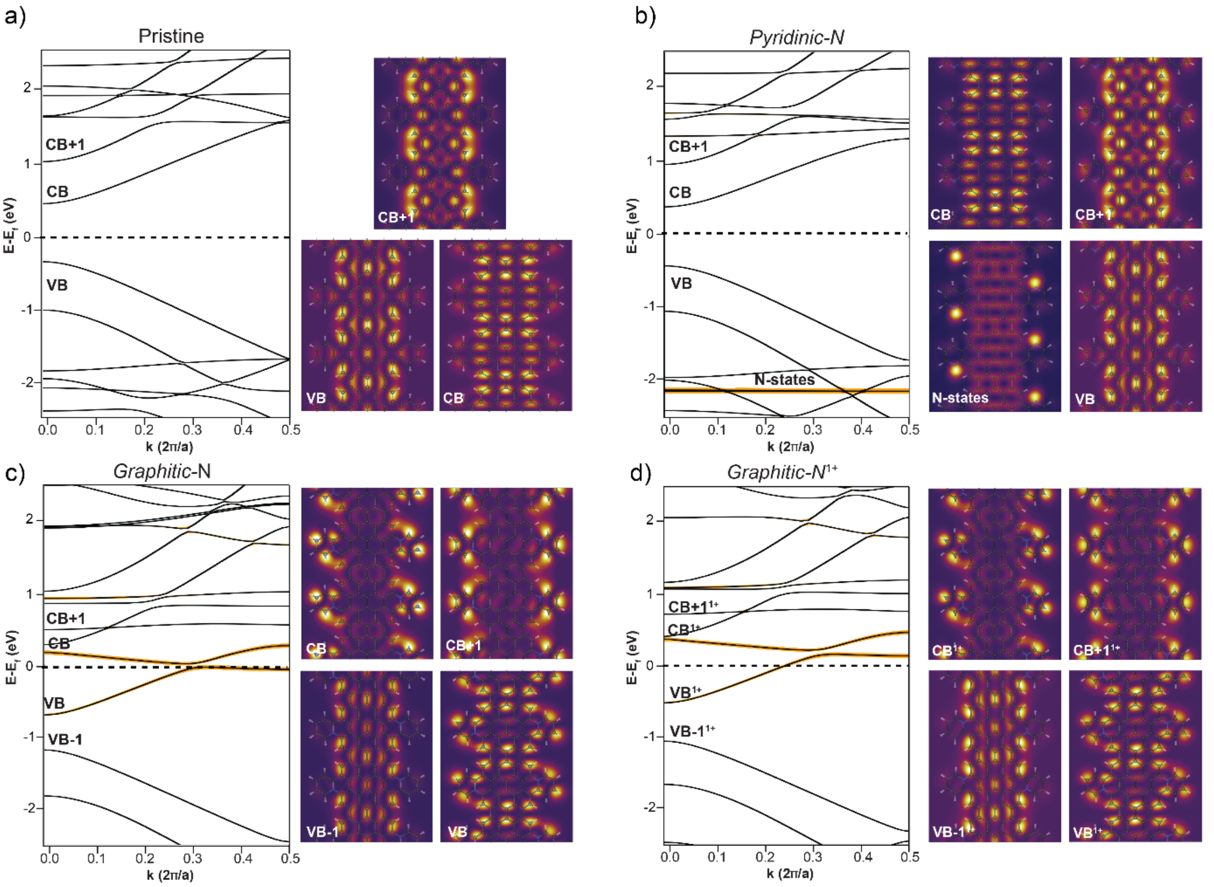

Figure SI 13: Band structure and corresponding PDOS maps of selected bands. Band structures and DOS maps of the frontier energy levels evaluated at the Γ-point of different Py-7-AGNR structures: a) pristine b) *pyridinic*-N c) *graphitic*-N and d) *graphitic*-N^{1+}. The corresponding bands are reported in each panel. Pristine and *pyridinic*-N geometries have very similar properties, with almost the same band gap. The main effect of the N is a localized state below -2 eV. Contrary, the charge-neutral *graphitic*-N case is different. Thanks to the additional electrons from the N atoms contributed to the π-electron system, the band forming the (empty) CB of the pristine Py-7-AGNR structure is now occupied. This shifts the Fermi level E_f and the system has a small gap. *Graphitic-N^{1+}* was modelled by removing one electrons per unit cell for comparison to the experimental results. This partial transfer empties the newly occupied level and shifts the E_f so that it crosses the VB resulting in half-filled band.

Supplementary Information Methods

Au(111) single crystal surfaces (MaTeck GmbH) were prepared by iterative Ar^+ sputtering and annealing cycles. Prior to sublimation of molecules, the surface structure and cleanliness was checked by STM imaging. The molecular precursors DP-DPBA were filled into quartz crucibles of a home-built evaporator and sublimed at around 300 °C to achieve a rate of 0.45 Å min^{-1} onto the single crystal surfaces held at room temperature. STM and AFM measurements were performed with a commercial low-temperature STM/AFM from Scienta Omicron operated at a temperature of 4.5 K (LHe) and base pressure below 5×10^{-11} mbar. STM images were acquired in constant-current mode (overview and high-resolution imaging), *dI/dV* spectra and *dI/dV* maps were acquired in constant-height mode. Differential conductance *dI/dV* spectra and maps were obtained with a lock-in amplifier. dI/dV parameters are reported in the figure captions. Bond-resolved nc-AFM images were acquired in constant-height mode with CO-functionalized tips at low bias voltages while recording frequency and current signals. Open feedback parameters and lowering of the tip height Δz are reported in the figure caption. The data were processed with Wavemetrics Igor Pro software.

DFT calculations were executed using the AiiDAlab[2] applications, based on AiiDA[3] workchains designed for the DFT code CP2K[4] (systems adsorbed on gold) and for the DFT code Quantum Espresso[5] (bandstructure calculations). Surface-adsorbate setups were modeled within a periodic slab scheme. The simulation cell included four Au atomic planes along the [111] orientation. Hydrogen atoms passivated one face of the slab to mitigate Au(111) surface states. A 40 Å vacuum layer was included to isolate the system from its periodic images along the axis orthogonal to the slab. Electronic wavefunctions were represented via TZV2P Gaussians basis sets for C, N, H, and DZVP for Au. Plane-waves basis set cutoff for the charge density was set at 600 Ry. Norm-conserving Goedecker–Teter–Hutter pseudopotentials were employed. The PBE GGA[6] approximation for the exchange correlation functional was used and Grimme's D3[7] van der Waals corrections were included. Au supercells varied in size depending on the adsorbate, ranging from 28.12 × 26.54 $Å^2$ (corresponding to 598 Au atoms) to 66.37 × 29.48 $Å^2$ (1538 Au atoms). Geometry optimizations were performed with the bottom two atomic planes constrained while relaxing others until forces were below 0.005 eV ˚A. For nc-AFM simulations, DFT equilibrium geometries and electrostatic potentials were used alongside Hapala's probe-particle code[8].

For the bandstructure calculations, ultrasoft pseudopotentials, from the SSSP[9] were employed to model the ionic potentials. A cutoff of 50 Ry (400 Ry) was used for the plane wave expansion of the wave functions (charge density). The simulation cell contained 15 Å of vacuum in the non-periodic directions to minimize interactions among periodic replicas of the system. The thickness of the vacuum region, the sampling of the BZ and the cutoff ensure convergency of the computed band structures. The atomic positions of the ribbon atoms and the cell dimension along the ribbon axis were optimized till forces were lower than 0.002 eV/A and the pressure in the cell was negligible. The band structures are aligned to the vacuum level computed from the average electrostatic potential in the vacuum region.

Supplementary Information References

[1] Xu, X., Di Giovannantonio, M., Urgel, J.I., Pignedoli, C.A., Ruffieux, P., Müllen, K., Fasel, R., Narita, A.: On-surface activation of benzylic C-H bonds for the synthesis of pentagon-fused graphene nanoribbons. *Nano Res.* **14**, 4754–4759 (2021).

[2] Yakutovich, A.V., Eimre, K., Schütt, O., Talirz, L., Adorf, C.S., Andersen, C.W., Ditler, E., Du, D., Passerone, D., Smit, B., et al.: Aiidalab an ecosystem for developing, executing, and sharing scientific workflows. *Computational Materials Science* **188**, 110165 (2021).

[3] Pizzi, G., Cepellotti, A., Sabatini, R., Marzari, N., Kozinsky, B.: Aiida: automated interactive infrastructure and database for computational science. *Computational Materials Science* **111**, 218–230 (2016).

[4] Hutter, J., Iannuzzi, M., Schiffmann, F., VandeVondele, J.: cp2k: atomistic simulations of condensed matter systems. *Wiley Interdisciplinary Reviews: Computational Molecular Science* **4**, 15–25 (2014).

[5] Giannozzi, P., Baroni, S., Bonini, N., Calandra, M., Car, R., Cavazzoni, C., Ceresoli, D., Chiarotti, G.L., Cococcioni, M., Dabo, I., et al.: Quantum espresso: a modular and open-source software project for quantum simulations of materials. *J. Phys. Condens Matter* **21**, 395502 (2009).

[6] Perdew, J.P., Burke, K., Ernzerhof, M.: Generalized gradient approximation made simple. *Phys. Rev. B* **54**, 16533 (1996).

[7] Grimme, S., Antony, J., Ehrlich, S., Krieg, H.: A consistent and accurate ab initio parametrization of density functional dispersion correction (DFT-D) for the 94 elements H-Pu. *J. Chem. Phys.* **132** (2010).

[8] Hapala, P., Kichin, G., Wagner, C., Tautz, F.S., Temirov, R., Jel ínek, P.: Mechanism of high-resolution STM/AFM imaging with functionalized tips. *Phys. Rev. B* **90**, 085421 (2014).

[9] Probert, M.I.J., Hasnip, P.J., Lejaeghere, K., Bihlmayer, G., Bjorkman, T., Blaha, P., Blum, V., Caliste, D., Castelli, I.E., Dal Corso, A., et al.: Reproducibility in density functional theory calculations of solids. *Science* **351**, 3000 (2016)
